# Validated names for experimental studies on race and ethnicity

**DOI:** 10.1038/s41597-023-01947-0

**Published:** 2023-03-10

**Authors:** Charles Crabtree, Jae Yeon Kim, S. Michael Gaddis, John B. Holbein, Cameron Guage, William W. Marx

**Affiliations:** 1 Assistant Professor, Department of Government, Dartmouth College, Hanover, 03755 USA; 2 Assistant Research Scholar, SNF Agora Institute, Johns Hopkins University, Baltimore, 21218 USA; 3 Senior Research Scientist, NWEA Research, Portland, OR, 97209; Associate Professor, Department of Sociology, University of California – Los Angeles, Los Angeles, 90095 USA; 4 Associate Professor, Frank Batten School of Leadership and Public Policy, University of Virginia, Charlottesville, 22904 USA; 5 Dartmouth College, Hanover, 03755 USA

**Keywords:** Sociology, Politics

## Abstract

A large and fast-growing number of studies across the social sciences use experiments to better understand the role of race in human interactions, particularly in the American context. Researchers often use names to signal the race of individuals portrayed in these experiments. However, those names might also signal other attributes, such as socioeconomic status (e.g., education and income) and citizenship. If they do, researchers would benefit greatly from pre-tested names with data on perceptions of these attributes; such data would permit researchers to draw correct inferences about the causal effect of race in their experiments. In this paper, we provide the largest dataset of validated name perceptions to date based on three different surveys conducted in the United States. In total, our data include over 44,170 name evaluations from 4,026 respondents for 600 names. In addition to respondent perceptions of race, income, education, and citizenship from names, our data also include respondent characteristics. Our data will be broadly helpful for researchers conducting experiments on the manifold ways in which race shapes American life.

## Background & Summary

The second decade of the 21st century has been marked with vivid and often violent evidence about the central role of race in American society: police killings of unarmed minorities, the rise of the Black Lives Matter movement and counter mobilizations, national debates about immigration policies targeting individuals from predominantly Muslim countries, and growing anti-Asian attitudes (to name a few). (We use ‘race’ as shorthand for both race and ethnicity throughout this paper. Social scientists often use them as synonyms, even if they treat the two concepts as substantively different^1–5^. For a critique of this approach, see Wimmer (2008)^6^.) Accordingly, a large, rapidly expanding literature across the sciences now uses experiments (i.e., conjoint, field and correspondence, and vignette) to better and more fully understand the causal effect of race on American attitudes, behaviors, and choices^7–10^. Evidence of this trend can be seen in Google Scholar search results for “racial discrimination” and “experiment,” which currently yields over 77,000 papers or books, 13,000 (or 17%) of which have been published or made available since 2020. Similarly, “conjoint” and “race” produces 55,000 results; “audit study” and “race” produces just under 5,000 results; and “list experiment” and “race” produces just under 1,000 results. (Search performed on October 24, 2022) These studies cover topics as broad and diverse as studies of the role of race in employment, housing, medicine, education, and government and public services^11^. In conjoint and vignette experiments, researchers typically present subject participants with a fictional situation and randomly vary the race of the individual it describes^12–15^. In correspondence and other field experiments, researchers often create fictional identities that they use to interact with subject participants^11,16–21^. In all of these experiments, researchers primarily use *names* to signal the racial identity of the fictitious individuals that they describe or create^9,17^, and thereby to estimate the effect of race on some attitude or behavior. (Other modes of manipulating race such as images or direct statements of one’s racial identity may be even more “bundled” treatments than names or provide incentives that elicit a very different response^22,23^.)

However, there are at least two potential problems with this approach. First, the names researchers use might not accurately signal the intended race^17,24^. In other words, the public’s racial perceptions of names might differ from researchers’ racial perceptions. This gap in perceptions could arise *even if* a name is drawn from administrative records (e.g., the Census) that predominantly is held by citizens of a specific race (as is customary in contemporary experiments). If citizens misperceive the racial cue, then subject participants might not ‘receive’ the treatment that researchers intend to deliver. This phenomena could cause racial treatment effects to be either attenuated or magnified—depending on the direction of the misperception and the reference category—and give the impression that race matters less or more to subject participants than it actually does.

Second, an equally consequential problem is that the names researchers use might not only signal race but also some other characteristics^17,25–27^. For example, imagine a survey experiment designed to assess anti-Asian attitudes. A stylized version might present a respondent with a vignette about a potential candidate for political office and randomize her name. Researchers could, for example, use the name ‘Hillary Clinton’ to indicate a White candidate and ‘Yuriko Koike’ to indicate an Asian candidate. Respondents may evaluate ‘Yuriko Koike’ as an Asian (or, more specifically, Japanese) person. However, they may also think that ‘Yuriko Koike’ is highly educated, earns a lot of income, or isn’t a U.S. citizen, among other possibilities. These non-racial signals complicate how researchers interpret the effect(s) of their Asian treatment because respondents may answer based on racial perceptions and other unintended signals^28,29^. A study may show that Asian names elicit less support than White names, but researchers cannot be certain *why* this is the case. Respondents may be less likely to support Asian candidates because of their race, but respondents may negatively react to some other characteristic that the name signals. More generally, if researchers intend to interpret the causal effects of name treatments to be about race and race alone, they need to select names across races that are equal or nearly equal across other orthogonal dimensions. The extent of this threat to inference depends on the research question one wants to answer. There may be certain circumstances where a bundled treatment may be of interest. After all, in the real-world, class, social attributes, and individual traits may combine to drive racial discrimination. As such, covarying treatments may not be threats to inference, but, rather, an important part of the story of racial discrimination^30^.

Despite these important considerations theoretically, there remains a gap between theory and practice here; indeed, many researchers do not measure perceptions of the names they use in their experiments either before or after fielding. Thus, researchers cannot be sure that they are signaling race appropriately and in isolation (if that is their desire). Additionally, scholars’ downstream inferences about the role of race in society may be misguided. While a select few studies provide researchers with validated names, they do so either (1) for first names only, which is problematic since last names provide crucial information on racial identity^17^ and are often used in experimental studies, or (2) for a very limited range of racial conditions^31,32^. In both cases, they provide a small number of names and limited attributes.

In this paper, we introduce three large datasets on name perceptions collected with independent samples of subjects (*n* = 1,004; 1,989; 1,033)^33,34^. The unit of analysis for these datasets is the name-evaluation, or a single respondent’s evaluation of a single name. Each dataset contains respondent evaluations of 600 names across four important dimensions: race, education level, income, and citizenship status. Two of these datasets also include respondent covariates, including their age, education level, gender, race, and citizenship status. Taken together, these datasets comprise over 44,170 subject participant name evaluations. We also provide a separate dataset that aggregates respondent perceptions to the name level and a R package—validatednamesr—and associated ﻿﻿https://jaeyk.github.io/validatednamesr/website ﻿﻿that assists researchers in their name selection.

Together, these resources contribute to the many scholars currently designing and conducting experiments on the manifold ways in which race shapes American life. It provides direct assistance to scholars by helping them proceed with greater clarity and direction in choosing the names that they use in their experiments. In so doing, this dataset provides the public service of assisting scholars to better understand the magnitude, scope, and potential mechanisms driving racial differences in our day to day lives.

## Methods

The name evaluations in this dataset were collected through three surveys that we fielded with U.S. residents in spring 2021. We conducted the first survey (hereafter Study 1) with 1,004 survey respondents via Prolific, an increasingly popular survey firm among social scientists^35^, on February 4, 2021. We conducted the second survey (Study 2) with a national, quota-based (gender, age, region, race, ethnicity) sample of 1,989 Americans through Lucid Marketplace, an established and widely used survey provider^36^, from May 19-31 2021. Finally, we conducted the third survey (Study 3) with a quota-based (gender, age, region) sample of 1,033 Americans who identify as first- or second-generation immigrants through Lucid Marketplace from May 18-27, 2021. Table 1 summarizes these studies. Since Study 2 and 3 captured respondent information, we applied for and received Institutional Review Board approval for them at Dartmouth College (STUDY00032300 and STUDY00032301). Study 1 did not collect any information about respondents and is just a coding task. Some scholars note that Lucid has rising rates of inattentiveness^37,38^. As we describe below, we both use a two-step attention check to flag inattentive respondents.

**Table 1:** Description of studies.

Study	Respondents	Tasks	Date
Study 1	1,004	15	February 4, 2021
Study 2	1,989	10	May 19–31, 2021
Study 3	1,033	10	May 18–27, 2021

### Measuring name perceptions

In each survey, we presented respondents with a series of names and a set of questions about each name. Each name was randomly drawn from a list of 600 unique names (first and last name combinations) using a web service that we created. We asked respondents to evaluate each randomly assigned name on four different dimensions: race, citizenship status, income, and education. Specifically, we displayed a name to respondents and asked them the following questions:

- What would you think would be the race of a person with this name?
  Answer options: White, Black or African American, American Indian or Alaska Native, Asian or Pacific Islander, Hispanic, Other
- Would you think that a person with this name is a U.S. citizen?
  Answer options: Yes, No
- What type of income do you think a person with this name would make?
  Answer options: Low income (Less than $40,100), Middle income ($41,000 - $120,400), Upper income (More than $120,400)
- What would be the highest level of education you think person with this name has likely completed?
  Answer options: High school, Bachelor’s degree, Master’s degree, Ph.D. degree
  If respondents answered that they thought the person with the name displayed was’Asian or Pacific Islander’, they were asked the following additional question.
- What do you think would be the specific race or ethnicity of a person with this name?

Answer options: Asian Indian, Chinese, Filipino, Japanese, Korean, Vietnamese, Other Asian

**Fig. 1:**
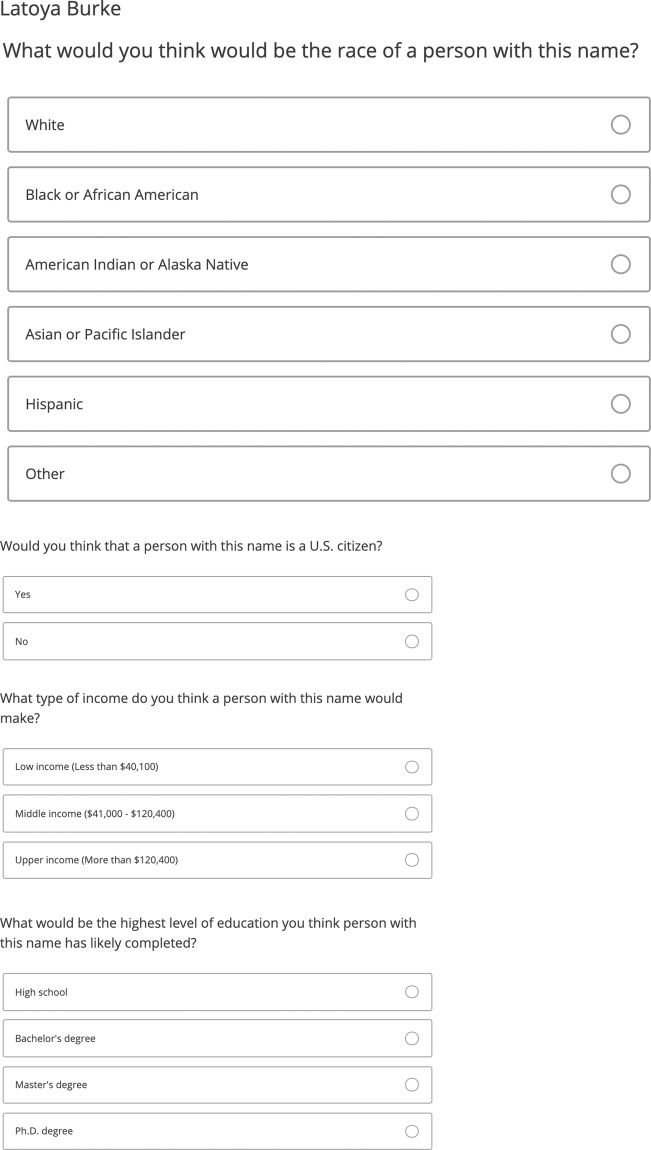
Example of a name task.

Respondents in Study 1 completed this name evaluation task 15 times. Respondents in Study 2 and Study 3 completed it 10 times. Figure 1 presents an example task.

### Name list creation

We used^39^ for our primary list of first names and^40^ for our list of last names. These are perhaps the two most authoritative lists of name prevalence in the United States containing racial associations. These datasets allow us to determine the 10 most frequently used, racially distinctive (or differentially expressed) first names and 10 most frequently used, racially distinctive last names among Whites, Blacks or African Americans, Asians or Pacific Islanders, and Hispanics. First, we subset the first name list to those that are ≥90% used by Asians or Pacific Islanders, Hispanics, and Whites, and ≥80% used by Blacks or African Americans. (We needed to use a lower threshold because there are fewer names that are differentially used by Blacks or African Americans compared to other races.) We then subset the last name list to include those that are ≥90% used by Asians or Pacific Islanders and Whites, ≥80% used by Hispanics, and ≥45% used by Blacks or African Americans. (Again, we needed to use a lower threshold because there are fewer names that are differentially used by Blacks or African Americans compared to other races.) We then created all permutations of first and last names for each racial condition, giving us 400 distinct names (10 × 10 × 4). Since we were also curious about how respondents would evaluate individuals with typically White first names and Asian last names, we used a third list of 10 English (or White) first names and last names commonly used by Chinese Americans^41^. (Chinese Americans, at 5.4 million, comprise 24% of the Asian population in the U.S. and are the largest Asian American origin group^42^.) We then created every permutation of these 20 first names and the 10 most commonly used names among Asian or Pacific Islanders, yielding an additional 200 (20 × 10) distinct names. These two combined lists yield 600 distinct names with racial associations.

### Respondent characteristics

In addition to capturing respondent perceptions of individual names, we also collected information about respondents in Study 2 and Study 3. Specifically, we asked respondents their age, gender, income, education level, race, and whether they are an American citizen. As we describe later in the ‘Usage Notes’ section, we believe that this additional information will be helpful to researchers for a variety of purposes.

### Intended racial identity

We append a separate dataset to these survey data that indicates what race each name is intended to signal based on the underlying prevalence data described above. For example, a name generated as a combination of first and last names commonly used by Hispanics is labeled ‘Hispanic’. There are four possible identities: Asian or Pacific Islander, Black or African American, Hispanic, and White. There is also a separate binary indicator for Asian identities with White first names.

## Data Records

With this paper, we provide four datasets. Table 2 describes the fields in study-1-names, study-2-names, and study-3-names. The unit of analysis for these datasets is the name-evaluation. study-1-names contains 14,935 observations, study-2-names contains 19,043, and study-3-names contains 10,192 observations.

**Table 2:** study-1-names, study-2-names, and study-3-names description of fields.

field	description	datasets
id	Character. Unique respondent ID.	All studies.
name	Character. Full name displayed to respondents.	All studies.
first	Character. First name displayed to respondents.	All studies.
last	Character. Last name displayed to respondents.	All studies.
w.asian	Binary indicator. Asian name with English (White) first name.	All studies.
identity	Character. Intended racial signal based on unique prevelance.	All studies.
race	Character. Respondent perceived race.	All studies.
specific.race	Character. Respondent perceived specific Asian race.	All studies.
citizen	Binary indicator. Whether respondents thought name belonged to a citizen.	All studies.
education	Character. Respondent perceived education.	All studies.
education.ord	Ordinal. Respondent perceived education.	All studies.
income	Character. Respondent perceived income.	All studies.
income.ord	Ordinal. Respondent perceived income.	All studies.
correct	Binary indicator. Whether respondents perceived intended race.	All studies.
res.age	Numeric. Respondent age.	Study 2, 3.
res.male	Binary indicator. Whether respondent is male.	Study 2, 3.
res.income	Ordinal. Respondent income.	Study 2, 3.
res.edu	Ordinal. Respondent education.	Study 2, 3.
res.citizenship	Binary indicator. Whether a respondent was a citizen.	Study 2, 3.
res.race	Character. Respondent race.	Study 2, 3.

Table 3 describes the fields in names. The unit of analysis for this dataset is the name. names contains 600 observations, one for each name.

**Table 3:** names description of fields.

name	Character. Full name displayed to respondents.	All studies.
first	Character. First name displayed to respondents.	All studies.
last	Character. Last name displayed to respondents.	All studies.
w.asian	Binary indicator. Asian name with English (White) first name.	All studies.
identity	Character. Intended racial signal based on unique prevelance.	All studies.
mean.correct	Numeric. Mean of correct matches for respondent perceived race and intended race.	All studies.
sd.correct	Numeric. Standard deviation of correct matches for respondent perceived race and intended race.	All studies.

We provide all four datasets at the following three repositories: (1) *GitHub* https://github.com/jaeyk/validated_names (2022), (2) an *Open Science Foundation* (OSF) data repository^34^ 10.17605/OSF.IO/AHPVQ (2022), and (3) *Harvard Dataverse*^33^ 10.7910/DVN/LP4EAR (2022). The datasets are available in four individual.csv and.rds files and a combined.rds and.rda file.

## Technical Validation

We take several steps to ensure that our data are high quality. One potential concern for data users here would respondent attentiveness and engagement. The general intuition is that inattentive respondents or ‘satisficers’—those who speed through the survey^43^—might answer randomly, introducing noise to our measures of name characteristics. We dealt with this in three ways. First, we recruited one of our samples through Prolific, which is thought to supply highly attentive survey respondents^44^. Second, we create a measure of attentiveness in the surveys we fielded with Lucid Marketplace. These surveys followed best practices and used a two-step attention check to screen out inattentive respondents^37,38^. Our Attentive measure codes respondents who completed both attention check questions successfully as ‘1’ and ‘0’ otherwise. Third, we inspect survey completion times and construct an indicator for ‘satisficing’, in line with Lucid’s recommendations. Our Satisficing measure codes respondents who completed the survey in less than 40% of the median completion time as ‘1’ and ‘0’ otherwise. While we provide data for all respondents who completed our surveys, we suggest that data users might want to consider subsetting our data to include only respondents who are attentive and were not satisficing. We think that this will help them decrease measurement error in their experimental treatments.

The second step we take is to formally assess the construct validity of our perceptions measures. We do this by estimating a set of ordinary least-squares models - four models for each of our three samples and another four for a pooled sample. Each model takes one of four different dependent variables and includes binary indicators for the four intended races — which we denote via shorthand as *Black*, *Hispanic*, *Asian*, and *White* — and whether a name is meant to signal an Asian with a White first name — *Asian (White first name)*. One dependent variable, *Correct*, is coded as ‘1’ if a respondent associates a name with the intended racial group and ‘0’ otherwise. The other three dependent variables are *Citizen* (0/1), *Education* (1-4), and Income *1*-*3*. Robust standard errors are calculated using the HC3 estimator. The estimation model is specified below:

$$Y={\beta }_{0}+{\beta }_{1}{\rm{Binary}}\;{\rm{indicator}}\;{\rm{for}}\;{\rm{the}}\;{\rm{four}}\;{\rm{intended}}\;{\rm{races}}+{\beta }_{2}{\rm{Asian}}\;{\rm{with}}\;{\rm{a}}\;{\rm{White}}\;{\rm{first}}\;{\rm{name}}+{\rm{error}}$$

**Fig. 2:**
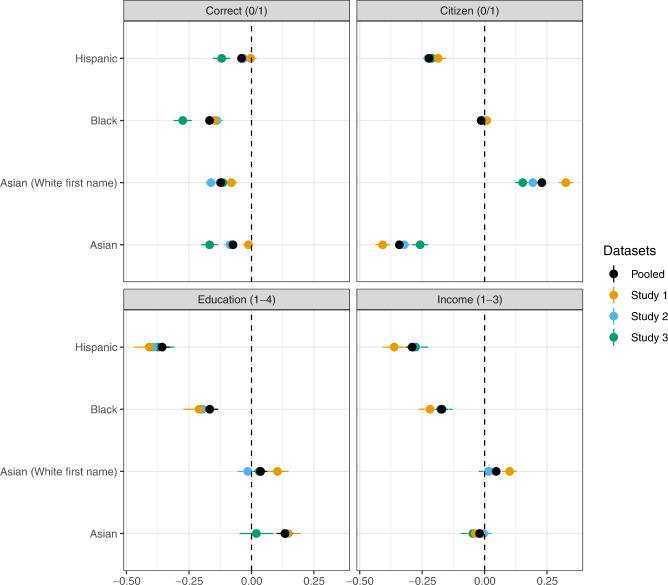
Results from four OLS models designed to assess data validity.

Figure 2 shows the results from these models. Plotted points are estimated coefficients (${\widehat{\beta }}_{1}$ and ${\widehat{\beta }}_{2}$) and bars are 95% confidence intervals. We can use these results to assess the general face validity of respondent perceptions. Starting with the top-right plot, we see that respondents from all samples think that people with our Hispanic names are roughly 20 percentage points less likely to be citizens (compared to White names). This belief is (unfortunately) in line with American views, which tend to conflate Hispanic identity and immigrant status^45^. Moving down the top-right plot, we also see that respondents from all samples think that individuals with English (or White) first names and Asian last names are more likely to be citizens than individuals who have both Asian first and last names. These findings align with anecdotal evidence about some Asians adopting White-sounding first names because Asian-sounding names are often associated with foreignness^46,47^.

Moving to the bottom plot, we also see that respondents from all samples believe that individuals with names intended to signal Hispanic or Black and African American status completed less education and made less money. This is indicated by the negative and statistically significant coefficients for the *Hispanic* and *Black and African American* coefficients across all samples. These results accord with the fact that, largely due to systemic racism in the U.S.^48,49^, individuals from those groups typically complete college at lower rates and earn less income than White people^50,51^.

**Fig. 3:**
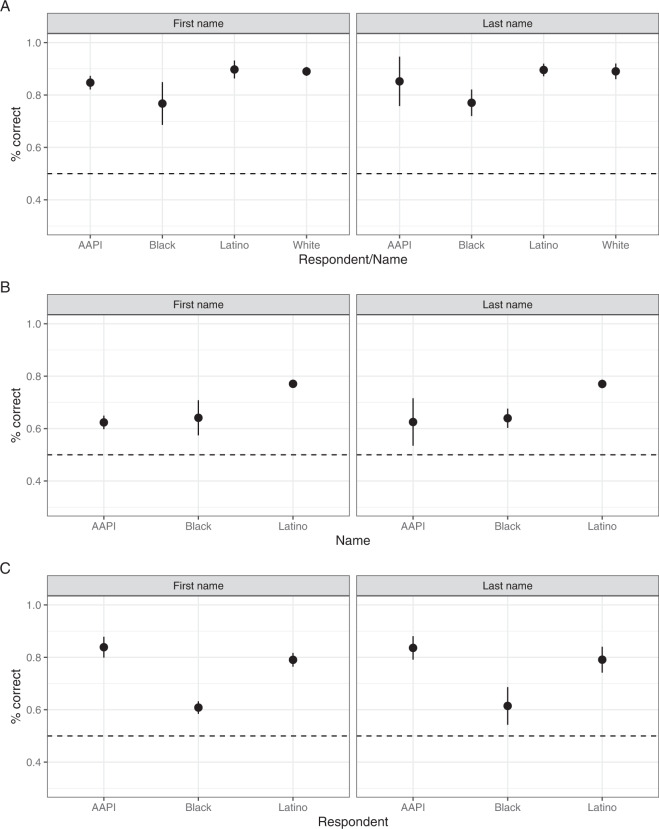
Within and between group assessment. (**A**): Within group assessment: a group member assesses their own group’s names, (**B**): Between group assessment (1), A white assesses other racial group members’ names, (**C**): Between group assessment (2), A non-white assesses whites’ names.

In addition, we validated how respondents assessed the intended race of names differently depending on whether they assessed their own group members’ names (within-group assessment) or their out-group members’ names (between-group assessment) (see Fig. 3). For the estimation, we used the same model above. In the figure, the dots represent the estimated coefficients of binary indicators for the four intended races. The bars represent 95% confidence intervals. On average, we find that respondents did a better job correctly identifying race from the names of their group members (85%) than from their out-group members (71%) (Panel A). Interestingly, on average, non-whites were more capable of correctly identifying whites’ names (75%) (Panel C) compared to the degree to which whites could do that with non-white names (68%) (Panel B). As Du Bois proposed in his argument on double consciousness^52^, in a racially hierarchical society, oppressed minorities are more likely to be informed about the dominant group than the other way around.

Taken together, these results suggest that subject participants were attentive, took the evaluation task seriously, and provided quality responses that align with existing findings.

Finally, we note that in Table S1 in the Supplementary Materials we provide the average and variance of the characteristics that we use for all 600 individual names tested. These provide researchers with a long list of names to use and match based on their research question of interest. In validating this list, a few things are clear. First, it is very difficult to find names that are perceived to be all to belong to the racial/ethnic group to which researchers assign them. For the Asian category, the highest perceived Asian name (Wei Li) is perceived as Asian by 91% of the population (SE=3.5%). For the Black condition the highest alignment goes to Lakisha Jackson (89.1% correct; SE=4.6%). For Hispanic, the most often perceive Hispanic name is a tie between Julio Perez (87.5% correct; SE=4.5%) and Guadalupe Rodriguez (87.5% correct; SE=4.2%). For the white category, the most white perceived name is Thomas Wagner (93.1%; SE=2.5%). These all have a high chance of being perceived as belonging to the condition that researchers may assign them; but none of these quantities is 100%. This suggests something that has not been appreciated by researchers previously; that name-based racial assignment is fuzzy. Most name-based studies—not measuring perceptions—miss out on this fact. Second, Table S1 validates the point that one must choose race-based names with great care. If one wanted to answer the question of race, independent of perceived citizenship, education, and income levels, it would not be appropriate, for example, to simply use the most racially distinct names listed above as these names have a great deal of divergence in terms of their perceived citizenship, education, and income. (If, however, one were interested in citizenship, education, and income as potential mechanisms, using racially distinctive names would be more defensible.) Finally, Table S1 validates that each of the perceived names has a distribution of perceptions. Some names may have a wider distribution on all of the dimensions perceived. For example, in the correct perception category, the standard errors range from 2.5% to 7.3%. In choosing one’s names for a study of interest it may be important to not only consider averages, but distributions. For example, if one is interested in dialing up the racial/ethnic signal, they may desire to choose a name with a high mean and low standard error—i.e. a name that many people perceive as belonging to that racial/ethnic and for whom there is relatively little disagreement on/variance in that categorization.

## Usage Notes

Researchers can use these names to signal an individual’s specific racial identity in a range of experimental tasks, such as survey experiments, conjoint experiments, and correspondence audits. They can do this by using the names in vignettes, conjoint tables, or mail and email requests commonly used in audits. Doing so will help researchers with the information equivalence problem fundamental to studies signalling race, and name-based studies specifically^26,27^.

There are at least three things that researchers should consider when using these names. First, they should decide whether they want to select names that vary across race but are constant across other perceived attributes *or* names that vary across race and other perceived attributes. Substantively, the choice depends on whether researchers want to examine the effect of race, holding other attributes constant and potentially partialing out or controlling for the effect of race, or whether they want to examine the effect of race, allowing other attributes *likely influenced by race* to vary.

Second, researchers should seriously consider using not just one but several names to indicate a particular race in their studies^31^. If researchers use a single name as a particular racial treatment, they cannot be sure that any effects of race they estimate are not limited to the one selected name. If researchers, on the other hand, randomly assign one of several names as a particular racial treatment, then they can better ensure that their race estimates are robust to a range of names.

Third, while we have focused on using these names to signal racial identities, researchers can also use the provided datasets to create experimental treatments that signal differences in citizenship, education, and income both within and across races. Using names in this manner might be particularly useful in contexts where it is sometimes difficult to explicitly state these attributes, such as in audit experiments. Researchers can also use the names in these datasets to explore possible interactions between race and other attributes. For example, scholars might use our names to investigate theories about how the effect of race might be conditional on perceived education, income, or citizenship.

Fourth, researchers can use the respondent demographic attributes collected in Study 2 and Study 3 to understand how racial perceptions might vary across groups. This is useful information if researchers are interested in conducting their experiments with specific types of people, such as less-educated Whites. The general idea is that researchers can examine the perceptions of respondents that most closely match their intended samples and use these data to select name treatments.

We are making our datasets available under the Creative Commons Attribution 4.0 International License, which grants licensees the right to use, share, and adapt the datasets if they agree to attribute the archive and place no further restrictions on its use. In line with^53^, we also make a non-binding request that researchers contact the authors with information about any publicly available working papers or publications that they produce with these names, so we can include an updated usage list in our data archive.

## Supplementary information

(Supplementary) Supporting Information

## Data Availability

Our datasets and the technical validation results are available as part of our data archive on (1) *GitHub* https://github.com/jaeyk/validated_names (2022), (2) *OSF*^34^ 10.17605/OSF.IO/AHPVQ (2022), and (3) *Harvard Dataverse*^33^ 10.7910/DVN/LP4EAR (2022). The archived GitHub repository (replication code) is available on *zenodo*^54^ 10.5281/zenodo.7460488 (2022).
