# Supplementary material for: Validated names for experimental studies on race and ethnicity
Source: Sci Data. 2023 Mar 10;10:130. doi: 10.1038/s41597-023-01947-0 (PMC10006241; doi:10.1038/s41597-023-01947-0)
Supplement: Supplementary file 1 — (Supplementary) Supporting Information [file 41597_2023_1947_MOESM1_ESM.pdf]

Supplemental Information: Validated Names for Experimental Studies on Race and Ethnicity

Charles Crabtree[1,†*], Jae Yeon Kim[2,†], S. Michael Gaddis[3,†], John B. Holbein[4,†], Cameron Guage[5,†], and William W. Marx[5,†]

[1]Assistant Professor, Department of Government, Dartmouth College, Hanover, 03755, USA
[2]Assistant Research Scholar, SNF Agora Institute, Johns Hopkins University, Baltimore, 21218
[3]Senior Research Scientist, NWEA Research, Portland, OR, 97209; Associate Professor, Department of Sociology, University of California – Los Angeles, Los Angeles, 90095, USA
[4]Assistant Professor, Frank Batten School of Leadership and Public Policy, University of Virginia, Charlottesville, 22904
[5]Dartmouth College, Hanover, 03755, USA
[*]corresponding author(s): Charles Crabtree (crabtree@dartmouth.edu)

ABSTRACT

Table S1: Average Characteristics for Individual Names, Sorted by Identity and Probability of a Correct Response

Name	Identity	Pr. Correct	Pr. Citizen	Income (1-3)	Education (1-4)	Citizen SE	Income SE	Education SE	Correct SE	N
Wei Li	Asian	0.910	0.493	1.970	2.313	0.0615	0.0823	0.113	0.0351	67
Hung Chen	Asian	0.891	0.609	2.078	2.312	0.0615	0.0781	0.128	0.0393	64
Hung Wang	Asian	0.882	0.485	2.103	2.441	0.0611	0.0815	0.123	0.0394	68
Jian Li	Asian	0.882	0.553	2.013	2.197	0.0574	0.0761	0.0992	0.0373	76
May Chen	Asian	0.878	0.789	2.067	2.371	0.0433	0.0648	0.100	0.0347	90
Hong Wang	Asian	0.871	0.571	1.929	2.329	0.0596	0.0847	0.122	0.0403	70
Li Chen	Asian	0.867	0.617	2	2.283	0.0633	0.0713	0.117	0.0443	60
Young Le	Asian	0.864	0.727	2.045	2.288	0.0552	0.0627	0.0857	0.0426	66
Young Li	Asian	0.862	0.600	2.215	2.415	0.0612	0.0775	0.109	0.0432	65
Wei Yang	Asian	0.855	0.594	2.058	2.493	0.0595	0.0769	0.123	0.0427	69
Jin Chen	Asian	0.853	0.588	1.985	2.309	0.0601	0.0798	0.119	0.0433	68
Thanh Wang	Asian	0.852	0.689	2.033	2.344	0.0598	0.0822	0.117	0.0458	61
Hung Kim	Asian	0.851	0.541	1.986	2.189	0.0583	0.0680	0.107	0.0416	74
Alan Wang	Asian	0.851	0.878	2.110	2.260	0.0383	0.0718	0.103	0.0416	74
Dan Yang	Asian	0.848	0.773	2.091	2.545	0.0520	0.0770	0.108	0.0445	66
Andy Wang	Asian	0.845	0.817	2.085	2.324	0.0462	0.0688	0.0998	0.0432	71
Jin Yang	Asian	0.841	0.623	2.101	2.362	0.0588	0.0720	0.107	0.0444	69
Hung Yang	Asian	0.835	0.557	1.974	2.291	0.0562	0.0706	0.102	0.0420	79
Jin Wang	Asian	0.835	0.709	2	2.443	0.0514	0.0598	0.0949	0.0420	79
Jin Le	Asian	0.833	0.611	2.028	2.380	0.0579	0.0625	0.0990	0.0442	72
Jin Kim	Asian	0.833	0.643	1.976	2.286	0.0526	0.0586	0.0840	0.0409	84
Amy Li	Asian	0.831	0.859	2.028	2.254	0.0416	0.0722	0.111	0.0448	71
Li Tran	Asian	0.831	0.662	1.986	2.296	0.0565	0.0680	0.0949	0.0448	71
Grace Yang	Asian	0.831	0.847	2.153	2.627	0.0472	0.0718	0.123	0.0493	59
Amy Yang	Asian	0.830	0.830	2.057	2.509	0.0521	0.0827	0.116	0.0521	53
May Yang	Asian	0.829	0.671	1.957	2.343	0.0565	0.0718	0.117	0.0454	70
Hong Pham	Asian	0.826	0.465	1.942	2.282	0.0541	0.0648	0.101	0.0412	86
Grace Chen	Asian	0.824	0.879	2.022	2.297	0.0344	0.0563	0.0904	0.0401	91
Hong Chen	Asian	0.823	0.532	2.038	2.215	0.0565	0.0661	0.0965	0.0432	79
Peter Li	Asian	0.822	0.836	2.219	2.507	0.0437	0.0654	0.103	0.0451	73
Jian Yang	Asian	0.821	0.628	2.065	2.423	0.0551	0.0675	0.113	0.0437	78
Yong Pham	Asian	0.820	0.607	2.033	2.213	0.0631	0.0809	0.119	0.0496	61
Yong Kim	Asian	0.819	0.667	1.972	2.236	0.0559	0.0655	0.104	0.0456	72
Hong Li	Asian	0.816	0.526	2	2.289	0.0577	0.0621	0.0952	0.0448	76
Hong Le	Asian	0.816	0.539	1.974	2.421	0.0576	0.0675	0.101	0.0448	76
Thanh Li	Asian	0.812	0.588	2.025	2.278	0.0554	0.0665	0.0969	0.0439	80
Jian Wang	Asian	0.811	0.568	2.041	2.342	0.0580	0.0731	0.0960	0.0458	74
Minh Le	Asian	0.810	0.646	1.987	2.205	0.0542	0.0662	0.0937	0.0444	79
Thanh Le	Asian	0.808	0.493	1.890	2.110	0.0589	0.0718	0.111	0.0464	73

Table S1: Average Characteristics for Individual Names, Sorted by Identity and Probability of a Correct Response

Name	Identity	Pr. Correct	Pr. Citizen	Income (1-3)	Education (1-4)	Citizen SE	Income SE	Education SE	Correct SE	N
Li Li	Asian	0.806	0.581	1.968	2.230	0.0632	0.0919	0.131	0.0506	62
Jane Li	Asian	0.806	0.821	2.119	2.418	0.0472	0.0690	0.107	0.0487	67
Wei Le	Asian	0.806	0.418	1.925	2.179	0.0607	0.0805	0.106	0.0487	67
Minh Yang	Asian	0.806	0.582	1.970	2.284	0.0607	0.0778	0.116	0.0487	67
Cindy Li	Asian	0.803	0.836	2.033	2.153	0.0478	0.0822	0.102	0.0513	61
Jane Yang	Asian	0.803	0.852	2.066	2.383	0.0458	0.0734	0.109	0.0513	61
Jenny Li	Asian	0.803	0.909	2.061	2.409	0.0357	0.0566	0.0915	0.0493	66
Li Nguyen	Asian	0.803	0.671	2	2.250	0.0543	0.0701	0.111	0.0460	76
Eric Wang	Asian	0.797	0.851	2.135	2.452	0.0416	0.0702	0.103	0.0471	74
Minh Chen	Asian	0.797	0.593	2.051	2.220	0.0645	0.0852	0.121	0.0529	59
Jian Pham	Asian	0.795	0.616	1.945	2.167	0.0573	0.0700	0.103	0.0476	73
Thanh Chen	Asian	0.795	0.562	2.068	2.315	0.0585	0.0685	0.103	0.0476	73
Young Wang	Asian	0.794	0.676	2.045	2.382	0.0572	0.0779	0.115	0.0494	68
Yong Li	Asian	0.793	0.655	2.103	2.368	0.0630	0.0766	0.122	0.0537	58
Minh Wang	Asian	0.792	0.558	1.905	2.184	0.0570	0.0749	0.105	0.0465	77
Minh Li	Asian	0.792	0.545	1.935	2.118	0.0571	0.0700	0.104	0.0465	77
May Wang	Asian	0.792	0.722	2.042	2.472	0.0532	0.0777	0.112	0.0482	72
Andy Yang	Asian	0.791	0.925	2.209	2.403	0.0323	0.0657	0.104	0.0500	67
Li Kim	Asian	0.790	0.630	2.038	2.346	0.0540	0.0699	0.0996	0.0455	81
Yong Wang	Asian	0.790	0.556	1.975	2.259	0.0556	0.0680	0.105	0.0455	81
Li Wang	Asian	0.789	0.645	2	2.333	0.0553	0.0710	0.114	0.0471	76
Yong Chen	Asian	0.788	0.515	1.955	2.167	0.0620	0.0663	0.100	0.0507	66
Jin Li	Asian	0.786	0.643	2.086	2.257	0.0577	0.0697	0.111	0.0494	70
Li Pham	Asian	0.783	0.683	1.833	2.233	0.0606	0.0862	0.135	0.0536	60
Alan Chen	Asian	0.782	0.795	2.090	2.462	0.0460	0.0711	0.107	0.0470	78
Albert Chen	Asian	0.782	0.839	2.057	2.379	0.0396	0.0700	0.0984	0.0446	87
Sam Yang	Asian	0.779	0.838	2.147	2.456	0.0450	0.0703	0.121	0.0507	68
Hong Yang	Asian	0.776	0.553	1.929	2.282	0.0542	0.0575	0.103	0.0455	85
Peter Chen	Asian	0.776	0.855	2.092	2.263	0.0406	0.0626	0.105	0.0481	76
Wei Kim	Asian	0.773	0.591	2.034	2.239	0.0527	0.0614	0.0996	0.0449	88
Dan Kim	Asian	0.773	0.879	2.061	2.492	0.0405	0.0804	0.110	0.0520	66
Alan Li	Asian	0.772	0.785	2.177	2.494	0.0465	0.0591	0.0899	0.0475	79
Yong Nguyen	Asian	0.771	0.543	2.014	2.300	0.0600	0.0747	0.114	0.0506	70
Thanh Yang	Asian	0.771	0.629	2.043	2.100	0.0582	0.0773	0.102	0.0506	70
David Yang	Asian	0.770	0.865	2.054	2.500	0.0400	0.0664	0.0948	0.0492	74
Young Chen	Asian	0.770	0.494	2.057	2.414	0.0539	0.0641	0.0975	0.0454	87
Hong Nguyen	Asian	0.770	0.598	2	2.218	0.0529	0.0749	0.103	0.0454	87
May Le	Asian	0.767	0.671	1.889	2.123	0.0554	0.0728	0.0994	0.0498	73
Amy Wang	Asian	0.767	0.767	1.945	2.205	0.0498	0.0700	0.103	0.0498	73

Table S1: Average Characteristics for Individual Names, Sorted by Identity and Probability of a Correct Response

Name	Identity	Pr. Correct	Pr. Citizen	Income (1-3)	Education (1-4)	Citizen SE	Income SE	Education SE	Correct SE	N
Jin Tran	Asian	0.767	0.667	1.933	2.237	0.0614	0.0667	0.114	0.0551	60
David Chen	Asian	0.764	0.875	2.099	2.417	0.0392	0.0831	0.122	0.0504	72
Andrew Wang	Asian	0.762	0.825	1.984	2.206	0.0482	0.0733	0.104	0.0541	63
Minh Kim	Asian	0.761	0.612	2.119	2.493	0.0600	0.0782	0.121	0.0525	67
Alice Chen	Asian	0.761	0.817	2.014	2.437	0.0462	0.0650	0.108	0.0510	71
Young Nguyen	Asian	0.761	0.634	1.958	2.155	0.0576	0.0648	0.104	0.0510	71
David Wang	Asian	0.760	0.907	2.067	2.413	0.0338	0.0693	0.0970	0.0496	75
Minh Nguyen	Asian	0.759	0.590	1.939	2.048	0.0543	0.0657	0.0889	0.0472	83
Thanh Kim	Asian	0.759	0.517	1.931	1.931	0.0662	0.0847	0.120	0.0567	58
Yong Tran	Asian	0.758	0.596	1.970	2.172	0.0496	0.0633	0.0850	0.0433	99
David Li	Asian	0.757	0.800	2.014	2.371	0.0482	0.0593	0.100	0.0516	70
Hung Li	Asian	0.756	0.654	2.038	2.359	0.0542	0.0784	0.105	0.0489	78
Jin Pham	Asian	0.756	0.674	2.012	2.082	0.0508	0.0608	0.0857	0.0466	86
Jane Wang	Asian	0.750	0.863	2.075	2.475	0.0387	0.0636	0.0889	0.0487	80
Vivian Yang	Asian	0.750	0.800	2.075	2.362	0.0450	0.0584	0.0962	0.0487	80
Eric Kim	Asian	0.750	0.842	1.947	2.224	0.0421	0.0698	0.0966	0.0500	76
Wei Tran	Asian	0.750	0.594	1.953	2.391	0.0619	0.0875	0.111	0.0546	64
Jian Chen	Asian	0.750	0.671	2.092	2.632	0.0543	0.0705	0.102	0.0500	76
Albert Wang	Asian	0.750	0.824	2.176	2.603	0.0466	0.0590	0.105	0.0529	68
Amy Le	Asian	0.747	0.797	2.076	2.329	0.0455	0.0692	0.0999	0.0492	79
Yong Yang	Asian	0.743	0.514	2.014	2.378	0.0585	0.0733	0.113	0.0511	74
Young Yang	Asian	0.742	0.591	1.970	2.061	0.0610	0.0747	0.103	0.0542	66
May Kim	Asian	0.742	0.758	2	2.092	0.0532	0.0716	0.0974	0.0542	66
Wei Wang	Asian	0.741	0.704	2.136	2.580	0.0511	0.0652	0.110	0.0490	81
Amy Chen	Asian	0.740	0.699	2.137	2.466	0.0541	0.0685	0.103	0.0517	73
Jane Le	Asian	0.739	0.797	2.014	2.362	0.0488	0.0838	0.109	0.0532	69
Peter Wang	Asian	0.739	0.855	2.261	2.493	0.0427	0.0763	0.0982	0.0532	69
Alan Yang	Asian	0.738	0.754	2.131	2.279	0.0556	0.0681	0.0995	0.0568	61
Jian Le	Asian	0.736	0.681	2.028	2.458	0.0553	0.0684	0.106	0.0523	72
Wei Chen	Asian	0.735	0.574	1.912	2.294	0.0604	0.0748	0.0984	0.0539	68
Jian Kim	Asian	0.735	0.662	1.912	2.353	0.0578	0.0686	0.0908	0.0539	68
Hong Kim	Asian	0.734	0.594	2.016	2.344	0.0619	0.0567	0.105	0.0556	64
Jane Kim	Asian	0.734	0.835	1.962	2.190	0.0420	0.0609	0.0902	0.0500	79
Hung Le	Asian	0.733	0.600	1.967	2.217	0.0638	0.0750	0.112	0.0576	60
Vivian Wang	Asian	0.730	0.730	1.968	2.317	0.0564	0.0597	0.106	0.0564	63
Andrew Le	Asian	0.730	0.851	2	2.216	0.0416	0.0617	0.0948	0.0520	74
Dan Nguyen	Asian	0.729	0.886	2.014	2.343	0.0383	0.0565	0.0973	0.0535	70
Albert Yang	Asian	0.729	0.829	2.086	2.343	0.0454	0.0667	0.106	0.0535	70
Alex Yang	Asian	0.729	0.843	2.086	2.486	0.0438	0.0807	0.109	0.0535	70

Table S1: Average Characteristics for Individual Names, Sorted by Identity and Probability of a Correct Response

Name	Identity	Pr. Correct	Pr. Citizen	Income (1-3)	Education (1-4)	Citizen SE	Income SE	Education SE	Correct SE	N
Andy Li	Asian	0.728	0.845	1.990	2.297	0.0359	0.0543	0.0862	0.0441	103
Cindy Yang	Asian	0.727	0.766	2.013	2.500	0.0485	0.0653	0.0982	0.0511	77
Dan Wang	Asian	0.727	0.727	2.046	2.231	0.0552	0.0708	0.113	0.0552	66
Alice Yang	Asian	0.726	0.871	2.131	2.403	0.0429	0.0792	0.0988	0.0571	62
Jian Tran	Asian	0.725	0.688	1.925	2.038	0.0521	0.0660	0.106	0.0502	80
Andrew Chen	Asian	0.725	0.800	2.050	2.241	0.0450	0.0687	0.0940	0.0502	80
Li Yang	Asian	0.725	0.609	1.899	2.203	0.0592	0.0776	0.114	0.0542	69
Andy Chen	Asian	0.725	0.884	2	2.304	0.0388	0.0715	0.0997	0.0542	69
Wei Nguyen	Asian	0.725	0.681	1.941	2.232	0.0565	0.0808	0.113	0.0542	69
Cecilia Wang	Asian	0.725	0.797	2.130	2.333	0.0488	0.0713	0.0961	0.0542	69
Young Kim	Asian	0.723	0.631	1.969	2.308	0.0603	0.0759	0.114	0.0559	65
Alice Wang	Asian	0.722	0.861	2.083	2.347	0.0410	0.0649	0.0992	0.0532	72
Jennifer Wang	Asian	0.722	0.875	2.167	2.556	0.0392	0.0740	0.0946	0.0532	72
Minh Pham	Asian	0.722	0.570	1.823	1.924	0.0561	0.0715	0.0878	0.0508	79
Cindy Wang	Asian	0.721	0.791	2.024	2.244	0.0441	0.0648	0.0905	0.0487	86
Jenny Chen	Asian	0.720	0.805	2.061	2.317	0.0440	0.0585	0.0900	0.0499	82
Hung Pham	Asian	0.718	0.676	1.887	2.197	0.0559	0.0682	0.115	0.0538	71
Jennifer Chen	Asian	0.718	0.974	2.218	2.526	0.0180	0.0595	0.0961	0.0513	78
Eric Yang	Asian	0.716	0.838	2.068	2.392	0.0431	0.0712	0.115	0.0528	74
Hong Tran	Asian	0.716	0.649	1.946	2.297	0.0559	0.0743	0.110	0.0528	74
Jenny Yang	Asian	0.716	0.811	2.041	2.365	0.0458	0.0622	0.105	0.0528	74
Sam Li	Asian	0.712	0.767	1.972	2.250	0.0498	0.0684	0.102	0.0533	73
Andrew Yang	Asian	0.711	0.928	2.329	2.610	0.0286	0.0627	0.0931	0.0501	83
Yong Le	Asian	0.710	0.507	2.014	2.265	0.0606	0.0758	0.104	0.0550	69
Jennifer Li	Asian	0.710	0.870	2.029	2.391	0.0408	0.0684	0.104	0.0550	69
Alex Chen	Asian	0.710	0.812	1.925	2.294	0.0474	0.0649	0.101	0.0550	69
Alice Li	Asian	0.709	0.886	2.063	2.570	0.0360	0.0658	0.0998	0.0514	79
Sam Le	Asian	0.708	0.847	2.069	2.403	0.0427	0.0722	0.111	0.0539	72
Eric Li	Asian	0.708	0.785	2.138	2.492	0.0514	0.0756	0.110	0.0569	65
Cindy Le	Asian	0.707	0.793	2.024	2.280	0.0450	0.0694	0.0906	0.0506	82
May Pham	Asian	0.707	0.773	1.893	2.176	0.0487	0.0646	0.0824	0.0529	75
Cecilia Yang	Asian	0.706	0.824	2.024	2.294	0.0416	0.0677	0.0944	0.0497	85
Young Pham	Asian	0.706	0.603	1.940	2.368	0.0598	0.0763	0.115	0.0557	68
Grace Li	Asian	0.701	0.866	2.104	2.239	0.0420	0.0710	0.111	0.0563	67
Eric Le	Asian	0.701	0.791	2.045	2.358	0.0500	0.0687	0.0968	0.0563	67
Andrew Li	Asian	0.701	0.828	2.057	2.388	0.0407	0.0619	0.0933	0.0494	87
Alex Wang	Asian	0.700	0.825	2.087	2.350	0.0427	0.0595	0.0960	0.0516	80
Vivian Chen	Asian	0.698	0.810	2.111	2.365	0.0499	0.0720	0.109	0.0583	63
Jian Nguyen	Asian	0.697	0.591	1.955	2.394	0.0610	0.0820	0.118	0.0570	66

Table S1: Average Characteristics for Individual Names, Sorted by Identity and Probability of a Correct Response

Name	Identity	Pr. Correct	Pr. Citizen	Income (1-3)	Education (1-4)	Citizen SE	Income SE	Education SE	Correct SE	N
Sam Chen	Asian	0.695	0.829	2.148	2.378	0.0418	0.0611	0.0976	0.0512	82
Grace Nguyen	Asian	0.694	0.833	2.014	2.236	0.0442	0.0727	0.104	0.0547	72
May Nguyen	Asian	0.693	0.760	1.933	2.173	0.0496	0.0719	0.108	0.0536	75
Jennifer Yang	Asian	0.693	0.795	2.011	2.375	0.0432	0.0656	0.0890	0.0494	88
Peter Yang	Asian	0.692	0.862	2.046	2.292	0.0432	0.0673	0.109	0.0577	65
Alex Li	Asian	0.691	0.721	2.044	2.265	0.0548	0.0677	0.0974	0.0564	68
Dan Li	Asian	0.688	0.838	2.101	2.388	0.0415	0.0613	0.0966	0.0521	80
Andrew Nguyen	Asian	0.686	0.829	2.029	2.116	0.0454	0.0643	0.0913	0.0559	70
May Li	Asian	0.685	0.630	2.014	2.260	0.0569	0.0689	0.0976	0.0547	73
Thanh Nguyen	Asian	0.679	0.577	1.936	2.128	0.0563	0.0715	0.0989	0.0532	78
Thanh Tran	Asian	0.676	0.577	1.886	1.972	0.0590	0.0721	0.100	0.0559	71
Young Tran	Asian	0.676	0.648	1.845	1.915	0.0571	0.0768	0.0935	0.0559	71
Sam Nguyen	Asian	0.676	0.811	2.122	2.378	0.0458	0.0692	0.105	0.0548	74
David Nguyen	Asian	0.675	0.753	1.987	2.260	0.0495	0.0728	0.116	0.0537	77
Jenny Kim	Asian	0.672	0.820	2.049	2.246	0.0496	0.0755	0.109	0.0606	61
Jenny Wang	Asian	0.672	0.766	2.172	2.556	0.0534	0.0724	0.113	0.0592	64
Cindy Chen	Asian	0.672	0.776	2.030	2.394	0.0513	0.0766	0.108	0.0578	67
Jin Nguyen	Asian	0.671	0.743	1.986	2.386	0.0526	0.0775	0.112	0.0565	70
Vivian Pham	Asian	0.671	0.849	1.986	2.315	0.0422	0.0661	0.0973	0.0554	73
Wei Pham	Asian	0.671	0.592	1.895	2.066	0.0567	0.0715	0.0943	0.0543	76
Sam Wang	Asian	0.671	0.734	2.114	2.333	0.0500	0.0720	0.110	0.0532	79
Dan Le	Asian	0.667	0.727	1.818	2.136	0.0552	0.0644	0.103	0.0585	66
Eric Nguyen	Asian	0.667	0.850	2.017	2.067	0.0465	0.0873	0.116	0.0614	60
Dan Chen	Asian	0.667	0.857	2.108	2.422	0.0384	0.0664	0.0909	0.0517	84
Hung Nguyen	Asian	0.667	0.500	1.767	2.034	0.0651	0.0836	0.118	0.0614	60
Alan Kim	Asian	0.662	0.794	2.132	2.397	0.0494	0.0809	0.109	0.0578	68
Vivian Li	Asian	0.659	0.753	2.012	2.435	0.0471	0.0567	0.0968	0.0517	85
Thanh Pham	Asian	0.658	0.532	1.797	1.962	0.0565	0.0729	0.0902	0.0537	79
Jane Chen	Asian	0.657	0.743	2.057	2.400	0.0526	0.0758	0.116	0.0571	70
Li Le	Asian	0.655	0.583	1.855	2.226	0.0541	0.0644	0.101	0.0522	84
Alice Le	Asian	0.653	0.847	1.903	2.028	0.0427	0.0599	0.103	0.0565	72
Jenny Tran	Asian	0.652	0.894	1.894	2.182	0.0382	0.0720	0.0939	0.0591	66
Alan Pham	Asian	0.650	0.825	1.950	1.949	0.0427	0.0614	0.0741	0.0537	80
Vivian Kim	Asian	0.649	0.905	1.986	2.176	0.0343	0.0680	0.0988	0.0559	74
Cecilia Li	Asian	0.648	0.803	2.085	2.282	0.0476	0.0688	0.0966	0.0571	71
Albert Nguyen	Asian	0.648	0.676	1.944	2.197	0.0559	0.0774	0.107	0.0571	71
Amy Nguyen	Asian	0.646	0.800	2.062	2.446	0.0500	0.0653	0.105	0.0598	65
Hung Tran	Asian	0.646	0.615	1.815	2	0.0608	0.0787	0.107	0.0598	65
Cindy Nguyen	Asian	0.646	0.772	1.886	2.190	0.0475	0.0624	0.107	0.0542	79

Table S1: Average Characteristics for Individual Names, Sorted by Identity and Probability of a Correct Response

Name	Identity	Pr. Correct	Pr. Citizen	Income (1-3)	Education (1-4)	Citizen SE	Income SE	Education SE	Correct SE	N
Eric Chen	Asian	0.645	0.790	2.048	2.339	0.0521	0.0777	0.100	0.0613	62
Peter Le	Asian	0.642	0.836	1.970	2.209	0.0456	0.0672	0.0987	0.0590	67
Peter Kim	Asian	0.632	0.868	2.149	2.441	0.0414	0.0713	0.0991	0.0589	68
Alex Kim	Asian	0.630	0.795	2.123	2.528	0.0476	0.0615	0.0906	0.0569	73
Cecilia Chen	Asian	0.629	0.823	2.210	2.419	0.0489	0.0732	0.102	0.0619	62
Andy Nguyen	Asian	0.629	0.857	1.971	2.257	0.0421	0.0545	0.0948	0.0582	70
Alice Nguyen	Asian	0.627	0.797	2	2.034	0.0529	0.0764	0.105	0.0635	59
Jennifer Kim	Asian	0.627	0.925	1.940	2.194	0.0323	0.0702	0.0957	0.0595	67
Andrew Kim	Asian	0.627	0.853	2.014	2.213	0.0411	0.0652	0.0916	0.0562	75
Alan Nguyen	Asian	0.626	0.824	2.066	2.187	0.0401	0.0621	0.0840	0.0510	91
Grace Le	Asian	0.625	0.859	2.031	2.381	0.0438	0.0738	0.107	0.0610	64
Alice Kim	Asian	0.623	0.855	2.101	2.304	0.0427	0.0658	0.0997	0.0588	69
Alan Le	Asian	0.621	0.782	1.966	2.337	0.0446	0.0528	0.0869	0.0523	87
Amy Kim	Asian	0.620	0.886	2.038	2.269	0.0360	0.0609	0.0960	0.0550	79
Dan Tran	Asian	0.620	0.870	1.935	2.099	0.0353	0.0459	0.0750	0.0509	92
David Le	Asian	0.619	0.810	2.016	2.317	0.0499	0.0767	0.110	0.0617	63
Alex Le	Asian	0.618	0.794	2.088	2.269	0.0494	0.0583	0.103	0.0594	68
Minh Tran	Asian	0.618	0.603	1.809	2.235	0.0598	0.0763	0.131	0.0594	68
Amy Pham	Asian	0.616	0.872	2.012	2.047	0.0362	0.0637	0.0825	0.0527	86
Jenny Le	Asian	0.615	0.862	1.906	2.185	0.0432	0.0658	0.0874	0.0608	65
Albert Li	Asian	0.614	0.814	2.057	2.271	0.0468	0.0785	0.111	0.0586	70
Yong Patel	Asian	0.612	0.627	1.970	2.164	0.0595	0.0863	0.114	0.0600	67
Cecilia Nguyen	Asian	0.612	0.847	1.965	2.271	0.0393	0.0614	0.0952	0.0532	85
Jenny Nguyen	Asian	0.605	0.765	2	2.272	0.0474	0.0689	0.0896	0.0547	81
Jane Nguyen	Asian	0.603	0.809	1.956	2.309	0.0480	0.0677	0.115	0.0598	68
Cindy Tran	Asian	0.600	0.963	2.087	2.263	0.0214	0.0539	0.0848	0.0551	80
Vivian Nguyen	Asian	0.600	0.787	2.040	2.270	0.0476	0.0696	0.108	0.0569	75
May Tran	Asian	0.600	0.812	1.847	2.095	0.0427	0.0615	0.0952	0.0535	85
Cecilia Pham	Asian	0.600	0.757	1.871	1.914	0.0516	0.0703	0.0949	0.0590	70
Cindy Kim	Asian	0.597	0.909	1.974	2.286	0.0330	0.0666	0.111	0.0563	77
Jennifer Le	Asian	0.597	0.889	2.097	2.208	0.0373	0.0744	0.0947	0.0582	72
Grace Kim	Asian	0.597	0.955	2.254	2.507	0.0255	0.0684	0.0936	0.0604	67
Jennifer Nguyen	Asian	0.592	0.855	2.053	2.368	0.0406	0.0673	0.0952	0.0567	76
Eric Tran	Asian	0.586	0.843	1.986	2.357	0.0438	0.0730	0.106	0.0593	70
Sam Kim	Asian	0.586	0.871	1.900	2.157	0.0403	0.0738	0.103	0.0593	70
Hong Patel	Asian	0.580	0.580	2.025	2.259	0.0552	0.0702	0.102	0.0552	81
Vivian Le	Asian	0.577	0.817	2.028	2.457	0.0462	0.0722	0.113	0.0590	71
Andrew Tran	Asian	0.574	0.926	2.015	2.176	0.0319	0.0611	0.115	0.0604	68
Cecilia Tran	Asian	0.571	0.873	2	2.290	0.0423	0.0716	0.0986	0.0628	63

Table S1: Average Characteristics for Individual Names, Sorted by Identity and Probability of a Correct Response

Name	Identity	Pr. Correct	Pr. Citizen	Income (1-3)	Education (1-4)	Citizen SE	Income SE	Education SE	Correct SE	N
Dan Pham	Asian	0.567	0.791	1.831	1.970	0.0500	0.0777	0.0876	0.0610	67
Cecilia Le	Asian	0.566	0.771	1.988	2.232	0.0464	0.0630	0.0955	0.0547	83
Peter Nguyen	Asian	0.565	0.768	1.957	2.162	0.0512	0.0784	0.110	0.0601	69
Albert Kim	Asian	0.564	0.795	2.038	2.333	0.0460	0.0669	0.0960	0.0565	78
David Kim	Asian	0.563	0.887	2.014	2.282	0.0378	0.0680	0.110	0.0593	71
Grace Wang	Asian	0.563	0.775	2.113	2.451	0.0499	0.0587	0.104	0.0593	71
Andy Kim	Asian	0.560	0.853	2.080	2.360	0.0411	0.0678	0.102	0.0577	75
Hung Patel	Asian	0.558	0.727	2.143	2.260	0.0511	0.0709	0.107	0.0570	77
Peter Tran	Asian	0.554	0.848	1.978	2.286	0.0377	0.0598	0.0846	0.0521	92
Albert Le	Asian	0.554	0.878	2.068	2.243	0.0383	0.0703	0.105	0.0582	74
Andy Le	Asian	0.551	0.872	2.039	2.154	0.0381	0.0726	0.100	0.0567	78
Sam Tran	Asian	0.550	0.825	1.886	2.075	0.0427	0.0624	0.0938	0.0560	80
Grace Tran	Asian	0.549	0.902	1.963	2.183	0.0330	0.0612	0.0816	0.0553	82
Alex Nguyen	Asian	0.544	0.779	1.912	2.239	0.0507	0.0654	0.107	0.0608	68
Peter Pham	Asian	0.536	0.870	1.884	2.116	0.0408	0.0760	0.108	0.0605	69
Alex Tran	Asian	0.529	0.912	1.897	1.956	0.0347	0.0633	0.0900	0.0610	68
Alex Pham	Asian	0.524	0.890	1.988	2.235	0.0347	0.0588	0.0982	0.0555	82
Jane Pham	Asian	0.523	0.841	1.852	2.068	0.0392	0.0637	0.0737	0.0536	88
David Tran	Asian	0.519	0.896	1.870	2.039	0.0350	0.0675	0.0847	0.0573	77
Wei Patel	Asian	0.519	0.778	2.074	2.404	0.0571	0.0910	0.130	0.0686	54
Eric Pham	Asian	0.517	0.883	1.900	2.300	0.0418	0.0740	0.112	0.0651	60
Vivian Tran	Asian	0.514	0.811	1.946	2.108	0.0458	0.0664	0.0992	0.0585	74
Sam Pham	Asian	0.513	0.842	1.960	2.092	0.0421	0.0696	0.101	0.0577	76
Alice Pham	Asian	0.506	0.864	1.962	2.123	0.0383	0.0602	0.0885	0.0559	81
Grace Pham	Asian	0.500	0.855	1.987	2.158	0.0406	0.0688	0.0938	0.0577	76
Amy Tran	Asian	0.500	0.903	2.069	2.153	0.0352	0.0694	0.0982	0.0593	72
Andrew Pham	Asian	0.500	0.782	1.821	2.013	0.0470	0.0747	0.102	0.0570	78
Alice Tran	Asian	0.500	0.883	2.017	2.283	0.0418	0.0651	0.104	0.0651	60
Albert Pham	Asian	0.493	0.821	1.940	2.062	0.0472	0.0597	0.100	0.0615	67
Andy Tran	Asian	0.488	0.829	1.902	2.329	0.0418	0.0663	0.103	0.0555	82
Albert Tran	Asian	0.488	0.854	2.025	2.259	0.0393	0.0608	0.0926	0.0555	82
Jin Patel	Asian	0.483	0.632	2.034	2.333	0.0520	0.0600	0.100	0.0539	87
Jenny Pham	Asian	0.479	0.836	1.890	1.986	0.0437	0.0718	0.102	0.0589	73
Jennifer Tran	Asian	0.476	0.905	1.937	2.302	0.0373	0.0548	0.110	0.0634	63
Li Patel	Asian	0.476	0.707	2.134	2.402	0.0506	0.0644	0.102	0.0555	82
Jennifer Pham	Asian	0.467	0.867	1.987	2.257	0.0395	0.0697	0.0924	0.0580	75
David Pham	Asian	0.462	0.877	1.938	2.154	0.0411	0.0689	0.101	0.0623	65
Cecilia Kim	Asian	0.458	0.875	1.875	2.125	0.0482	0.0924	0.125	0.0727	48
Jane Tran	Asian	0.456	0.924	1.974	2.115	0.0300	0.0657	0.0999	0.0564	79

Table S1: Average Characteristics for Individual Names, Sorted by Identity and Probability of a Correct Response

Name	Identity	Pr. Correct	Pr. Citizen	Income (1-3)	Education (1-4)	Citizen SE	Income SE	Education SE	Correct SE	N
Andy Pham	Asian	0.451	0.761	1.887	2.043	0.0510	0.0765	0.105	0.0595	71
Cindy Pham	Asian	0.449	0.795	1.870	2.090	0.0460	0.0700	0.0969	0.0567	78
Jian Patel	Asian	0.439	0.646	1.988	2.358	0.0531	0.0661	0.103	0.0551	82
Alan Tran	Asian	0.400	0.867	1.959	2.173	0.0395	0.0679	0.0836	0.0569	75
Minh Patel	Asian	0.373	0.667	2.027	2.440	0.0548	0.0735	0.116	0.0562	75
May Patel	Asian	0.362	0.787	2.076	2.316	0.0460	0.0669	0.112	0.0541	80
Thanh Patel	Asian	0.352	0.592	1.958	2.296	0.0588	0.0707	0.112	0.0571	71
Grace Patel	Asian	0.351	0.883	2.182	2.455	0.0369	0.0660	0.109	0.0547	77
David Patel	Asian	0.333	0.937	2.048	2.317	0.0310	0.0695	0.115	0.0599	63
Alex Patel	Asian	0.333	0.893	2.120	2.333	0.0359	0.0656	0.0917	0.0548	75
Andrew Patel	Asian	0.314	0.886	2.057	2.286	0.0383	0.0730	0.102	0.0559	70
Andy Patel	Asian	0.286	0.922	2.091	2.299	0.0307	0.0618	0.0907	0.0518	77
Eric Patel	Asian	0.284	0.881	2.030	2.418	0.0399	0.0704	0.105	0.0555	67
Dan Patel	Asian	0.278	0.903	2.139	2.319	0.0352	0.0665	0.0965	0.0532	72
Cindy Patel	Asian	0.273	0.948	2.026	2.390	0.0255	0.0739	0.0998	0.0511	77
Albert Patel	Asian	0.270	0.892	2.192	2.521	0.0363	0.0772	0.111	0.0520	74
Jennifer Patel	Asian	0.270	0.878	2.108	2.446	0.0383	0.0682	0.102	0.0520	74
Young Patel	Asian	0.267	0.640	1.920	2.053	0.0558	0.0729	0.100	0.0514	75
Vivian Patel	Asian	0.257	0.878	1.986	2.233	0.0383	0.0652	0.0985	0.0511	74
Cecilia Patel	Asian	0.250	0.817	2.017	2.433	0.0504	0.0770	0.107	0.0564	60
Sam Patel	Asian	0.232	0.879	2.061	2.253	0.0330	0.0571	0.0737	0.0427	99
Amy Patel	Asian	0.227	0.893	2.067	2.520	0.0359	0.0743	0.113	0.0487	75
Jenny Patel	Asian	0.222	0.914	2.074	2.413	0.0314	0.0675	0.111	0.0465	81
Alice Patel	Asian	0.219	0.877	2.123	2.329	0.0387	0.0702	0.0976	0.0488	73
Peter Patel	Asian	0.218	0.885	2.026	2.333	0.0364	0.0784	0.107	0.0470	78
Jane Patel	Asian	0.190	0.879	1.965	2.276	0.0431	0.0706	0.120	0.0519	58
Alan Patel	Asian	0.167	0.869	2.145	2.476	0.0370	0.0710	0.0965	0.0409	84
Lakisha Jackson	Black	0.891	0.957	1.739	1.587	0.0304	0.0903	0.101	0.0464	46
Tamika Jackson	Black	0.864	0.966	1.881	2.051	0.0238	0.0768	0.114	0.0450	59
Latasha Jackson	Black	0.852	0.984	1.754	1.885	0.0164	0.0864	0.102	0.0458	61
Lakisha Wood	Black	0.849	0.906	1.887	2.077	0.0405	0.0793	0.119	0.0496	53
Latoya Jackson	Black	0.831	0.972	1.943	1.930	0.0198	0.0811	0.103	0.0448	71
Tamika Williams	Black	0.815	0.938	1.831	2	0.0300	0.0713	0.0981	0.0485	65
Latonya Williams	Black	0.809	0.941	1.838	1.882	0.0287	0.0682	0.0877	0.0480	68
Latasha Burke	Black	0.806	0.919	1.806	1.902	0.0349	0.0721	0.104	0.0506	62
Tanisha Washington	Black	0.800	0.964	1.836	1.745	0.0255	0.0890	0.108	0.0544	55
Tamika Fox	Black	0.794	0.952	1.841	2	0.0270	0.0648	0.0876	0.0514	63
Latoya Burke	Black	0.790	0.952	1.855	1.968	0.0275	0.0757	0.115	0.0521	62
Lakisha Stone	Black	0.789	0.842	1.714	1.893	0.0487	0.0794	0.113	0.0545	57

Table S1: Average Characteristics for Individual Names, Sorted by Identity and Probability of a Correct Response

Name	Identity	Pr. Correct	Pr. Citizen	Income (1-3)	Education (1-4)	Citizen SE	Income SE	Education SE	Correct SE	N
Latasha Williams	Black	0.783	0.899	1.647	1.779	0.0366	0.0749	0.100	0.0500	69
Tanisha Williams	Black	0.778	0.907	1.815	1.870	0.0398	0.0652	0.0955	0.0571	54
Lakisha Williams	Black	0.768	0.986	1.725	1.942	0.0145	0.0741	0.107	0.0512	69
Latoya Washington	Black	0.764	0.927	1.727	1.964	0.0353	0.0606	0.107	0.0578	55
Ebony Jackson	Black	0.764	0.945	1.909	2.111	0.0309	0.0790	0.134	0.0578	55
Latonya Fox	Black	0.761	0.901	1.676	1.620	0.0356	0.0658	0.0812	0.0510	71
Tanisha Fox	Black	0.756	0.951	1.780	1.805	0.0239	0.0650	0.0822	0.0477	82
Tamika Wood	Black	0.750	0.929	1.821	1.839	0.0347	0.0810	0.107	0.0584	56
Lakisha Washington	Black	0.750	0.911	1.661	1.893	0.0385	0.0733	0.113	0.0584	56
Latasha Fox	Black	0.750	0.946	1.786	1.911	0.0304	0.0909	0.109	0.0584	56
Jermaine Wood	Black	0.746	0.984	1.778	1.762	0.0159	0.0766	0.0979	0.0553	63
Latonya Weaver	Black	0.746	0.881	1.712	1.847	0.0425	0.0766	0.105	0.0572	59
Jermaine Jackson	Black	0.742	0.955	1.879	1.955	0.0258	0.0850	0.121	0.0542	66
Ebony Williams	Black	0.742	0.968	1.839	2.065	0.0226	0.0734	0.108	0.0560	62
Tamika Burke	Black	0.730	0.937	1.778	1.984	0.0310	0.0731	0.103	0.0564	63
Latonya Washington	Black	0.730	0.932	1.730	1.905	0.0294	0.0728	0.102	0.0520	74
Lakisha Carpenter	Black	0.727	0.939	1.758	1.864	0.0296	0.0750	0.0987	0.0552	66
Latonya Stone	Black	0.727	0.939	1.652	1.831	0.0296	0.0732	0.0994	0.0552	66
Latonya Wood	Black	0.726	0.935	1.758	2	0.0315	0.0752	0.122	0.0571	62
Tanisha Jackson	Black	0.725	0.941	1.740	2.059	0.0333	0.0798	0.120	0.0631	51
Ebony Fox	Black	0.724	0.948	1.759	1.897	0.0293	0.0750	0.103	0.0592	58
Tamika Carpenter	Black	0.723	0.923	1.877	2.062	0.0333	0.0775	0.105	0.0559	65
Latasha Washington	Black	0.721	0.971	1.853	1.912	0.0206	0.0703	0.0977	0.0548	68
Tanisha Stone	Black	0.719	1	1.772	1.839	0	0.0868	0.116	0.0600	57
Latonya Jackson	Black	0.719	0.938	1.891	1.841	0.0305	0.0894	0.109	0.0566	64
Jermaine Williams	Black	0.719	0.891	1.812	1.953	0.0393	0.0625	0.105	0.0566	64
Lakisha Sullivan	Black	0.710	0.952	1.774	1.871	0.0275	0.0706	0.0875	0.0581	62
Lakisha Fox	Black	0.707	0.966	1.879	2.034	0.0242	0.0889	0.107	0.0603	58
Tamika Washington	Black	0.706	0.922	1.824	2.039	0.0380	0.0916	0.119	0.0644	51
Tanisha Carpenter	Black	0.706	0.961	1.765	2	0.0275	0.0953	0.101	0.0644	51
Latoya Fox	Black	0.700	0.883	1.733	1.983	0.0418	0.0819	0.127	0.0597	60
Ebony Washington	Black	0.697	0.934	1.829	1.961	0.0286	0.0685	0.0945	0.0530	76
Jermaine Sullivan	Black	0.697	0.894	1.848	1.864	0.0382	0.0755	0.0987	0.0570	66
Latasha Carpenter	Black	0.695	0.898	1.864	1.847	0.0397	0.0818	0.102	0.0605	59
Darnell Jackson	Black	0.694	0.919	1.839	2.049	0.0349	0.0803	0.111	0.0590	62
Darnell Williams	Black	0.692	0.954	1.877	2.031	0.0262	0.0775	0.114	0.0577	65
Lakisha Weaver	Black	0.691	0.909	1.727	1.800	0.0391	0.0798	0.108	0.0629	55
Latoya Weaver	Black	0.680	0.940	1.860	1.980	0.0339	0.0640	0.132	0.0666	50
Ebony Weaver	Black	0.679	0.929	1.821	1.891	0.0347	0.0887	0.112	0.0630	56

Table S1: Average Characteristics for Individual Names, Sorted by Identity and Probability of a Correct Response

Name	Identity	Pr. Correct	Pr. Citizen	Income (1-3)	Education (1-4)	Citizen SE	Income SE	Education SE	Correct SE	N
Ebony Stone	Black	0.673	0.982	1.764	1.927	0.0182	0.0819	0.103	0.0639	55
Latoya Williams	Black	0.667	0.947	1.842	1.947	0.0298	0.0822	0.124	0.0630	57
Latoya Wood	Black	0.667	0.947	1.693	1.787	0.0261	0.0735	0.0936	0.0548	75
Tanisha Weaver	Black	0.667	0.968	1.698	1.841	0.0223	0.0772	0.116	0.0599	63
Ebony Wood	Black	0.658	0.949	1.759	2	0.0248	0.0703	0.0936	0.0537	79
Latonya Carpenter	Black	0.656	0.906	1.873	1.969	0.0367	0.0767	0.109	0.0598	64
Tanisha Burke	Black	0.648	0.963	1.778	1.660	0.0259	0.0822	0.104	0.0656	54
Latoya Stone	Black	0.646	0.938	1.631	1.812	0.0300	0.0808	0.124	0.0598	65
Tamika Stone	Black	0.643	0.886	1.871	2.143	0.0383	0.0731	0.106	0.0577	70
Lakisha Burke	Black	0.639	0.861	1.732	1.889	0.0410	0.0694	0.106	0.0570	72
Latonya Burke	Black	0.633	0.917	1.733	1.700	0.0360	0.0784	0.0989	0.0627	60
Latasha Sullivan	Black	0.630	0.907	1.944	2.130	0.0398	0.0767	0.103	0.0663	54
Darnell Wood	Black	0.620	0.987	1.785	1.885	0.0127	0.0531	0.0894	0.0550	79
Latasha Weaver	Black	0.618	0.945	1.764	1.855	0.0309	0.0732	0.102	0.0661	55
Latonya Sullivan	Black	0.618	0.927	1.873	2	0.0353	0.0736	0.110	0.0661	55
Jermaine Fox	Black	0.618	0.909	1.855	2	0.0391	0.0838	0.118	0.0661	55
Jermaine Washington	Black	0.618	0.941	1.765	1.956	0.0287	0.0787	0.114	0.0594	68
Tamika Weaver	Black	0.617	0.933	1.850	2.050	0.0325	0.0884	0.113	0.0633	60
Tanisha Wood	Black	0.616	0.932	1.890	1.932	0.0298	0.0663	0.0922	0.0573	73
Darnell Fox	Black	0.615	0.954	1.906	2.031	0.0262	0.0762	0.103	0.0608	65
Ebony Burke	Black	0.609	0.906	1.844	2.078	0.0367	0.0779	0.114	0.0615	64
Jermaine Weaver	Black	0.609	0.938	1.891	1.906	0.0305	0.0775	0.0989	0.0615	64
Latasha Wood	Black	0.609	0.942	1.899	1.971	0.0283	0.0720	0.115	0.0592	69
Ebony Carpenter	Black	0.608	0.902	1.941	2.255	0.0421	0.0859	0.134	0.0690	51
Tamika Sullivan	Black	0.600	0.938	1.769	1.831	0.0300	0.0720	0.102	0.0612	65
Darnell Washington	Black	0.593	0.881	2.017	2.119	0.0425	0.0745	0.123	0.0645	59
Darnell Carpenter	Black	0.593	0.983	1.831	1.931	0.0169	0.0807	0.101	0.0645	59
Latoya Carpenter	Black	0.581	0.952	1.790	1.984	0.0275	0.0732	0.108	0.0632	62
Latasha Stone	Black	0.574	0.889	1.796	1.870	0.0432	0.0967	0.115	0.0679	54
Jermaine Burke	Black	0.574	0.882	1.879	1.971	0.0394	0.0666	0.0837	0.0604	68
Jermaine Carpenter	Black	0.556	0.952	1.857	2	0.0270	0.0779	0.106	0.0631	63
Tanisha Sullivan	Black	0.545	0.927	1.836	1.833	0.0353	0.0723	0.0980	0.0678	55
Ebony Sullivan	Black	0.541	0.918	1.902	2.033	0.0354	0.0800	0.126	0.0643	61
Darnell Stone	Black	0.533	0.967	1.831	2.050	0.0234	0.0807	0.107	0.0649	60
Darnell Weaver	Black	0.525	0.934	1.951	2.033	0.0320	0.0790	0.0991	0.0645	61
Jermaine Stone	Black	0.523	0.938	1.785	1.828	0.0300	0.0805	0.103	0.0624	65
Roosevelt Jackson	Black	0.491	0.964	1.891	2.036	0.0255	0.0924	0.135	0.0680	55
Latoya Sullivan	Black	0.491	0.909	1.855	1.889	0.0391	0.0838	0.108	0.0680	55
Darnell Burke	Black	0.481	0.909	1.883	2.078	0.0330	0.0715	0.107	0.0573	77

Table S1: Average Characteristics for Individual Names, Sorted by Identity and Probability of a Correct Response

Name	Identity	Pr. Correct	Pr. Citizen	Income (1-3)	Education (1-4)	Citizen SE	Income SE	Education SE	Correct SE	N
Darnell Sullivan	Black	0.426	0.885	1.934	2.066	0.0411	0.0696	0.0931	0.0638	61
Roosevelt Williams	Black	0.405	0.987	2.089	2.215	0.0127	0.0725	0.101	0.0556	79
Roosevelt Washington	Black	0.394	0.879	1.985	2.123	0.0405	0.0793	0.102	0.0606	66
Roosevelt Weaver	Black	0.357	0.971	2	2.114	0.0201	0.0788	0.118	0.0577	70
Roosevelt Burke	Black	0.351	0.930	2.070	2.429	0.0341	0.0745	0.116	0.0638	57
Roosevelt Wood	Black	0.339	0.949	1.881	1.966	0.0288	0.0768	0.108	0.0622	59
Roosevelt Fox	Black	0.283	0.943	2	2.038	0.0320	0.0893	0.114	0.0625	53
Roosevelt Stone	Black	0.269	0.896	2.030	2.433	0.0377	0.0795	0.128	0.0546	67
Roosevelt Carpenter	Black	0.262	0.820	2.131	2.393	0.0496	0.0792	0.108	0.0568	61
Roosevelt Sullivan	Black	0.229	0.917	2.042	2.292	0.0403	0.0940	0.130	0.0613	48
Julio Perez	Hispanic	0.875	0.696	1.691	1.768	0.0620	0.0773	0.114	0.0446	56
Guadalupe Rodriguez	Hispanic	0.875	0.656	1.688	1.766	0.0598	0.0767	0.106	0.0417	64
Julio Ramirez	Hispanic	0.873	0.673	1.709	1.745	0.0639	0.0848	0.117	0.0454	55
Javier Martinez	Hispanic	0.871	0.757	1.812	1.853	0.0516	0.0693	0.0966	0.0403	70
Salvador Gonzalez	Hispanic	0.868	0.642	1.679	1.755	0.0665	0.0751	0.107	0.0470	53
Jesus Ramirez	Hispanic	0.861	0.625	1.569	1.764	0.0575	0.0762	0.113	0.0410	72
Hector Rodriguez	Hispanic	0.860	0.605	1.674	1.690	0.0754	0.0863	0.134	0.0535	43
Alejandro Ramirez	Hispanic	0.857	0.714	1.698	1.619	0.0574	0.0836	0.102	0.0444	63
Hector Garcia	Hispanic	0.854	0.729	1.646	1.750	0.0648	0.0917	0.121	0.0515	48
Javier Sanchez	Hispanic	0.852	0.754	1.754	1.869	0.0556	0.0864	0.108	0.0458	61
Jesus Perez	Hispanic	0.850	0.650	1.650	1.683	0.0621	0.0851	0.102	0.0465	60
Hector Gonzalez	Hispanic	0.847	0.729	1.746	1.763	0.0584	0.0858	0.0977	0.0472	59
Julio Garcia	Hispanic	0.841	0.797	1.623	1.826	0.0488	0.0688	0.107	0.0444	69
Guadalupe Martinez	Hispanic	0.839	0.710	1.710	1.790	0.0581	0.0809	0.101	0.0471	62
Hector Ramirez	Hispanic	0.836	0.873	1.855	1.891	0.0454	0.0707	0.115	0.0503	55
Miguel Lopez	Hispanic	0.836	0.767	1.822	1.822	0.0498	0.0627	0.100	0.0437	73
Alejandro Perez	Hispanic	0.831	0.718	1.775	1.634	0.0538	0.0756	0.0925	0.0448	71
Juan Gonzalez	Hispanic	0.831	0.708	1.723	1.738	0.0569	0.0806	0.103	0.0469	65
Miguel Sanchez	Hispanic	0.830	0.736	1.792	1.849	0.0611	0.0779	0.116	0.0521	53
Jesus Martinez	Hispanic	0.828	0.759	1.810	1.793	0.0567	0.0833	0.109	0.0500	58
Jesus Lopez	Hispanic	0.828	0.690	1.707	1.793	0.0613	0.0887	0.125	0.0500	58
Jorge Rodriguez	Hispanic	0.827	0.731	1.712	1.788	0.0621	0.0839	0.124	0.0530	52
Julio Gonzalez	Hispanic	0.827	0.760	1.720	1.747	0.0496	0.0699	0.0872	0.0440	75
Alejandro Lopez	Hispanic	0.825	0.877	1.702	1.842	0.0439	0.0749	0.114	0.0508	57
Salvador Lopez	Hispanic	0.824	0.627	1.725	1.804	0.0684	0.0844	0.128	0.0539	51
Javier Gonzalez	Hispanic	0.821	0.731	1.701	1.836	0.0546	0.0767	0.101	0.0472	67
Salvador Ramirez	Hispanic	0.817	0.733	1.683	1.667	0.0576	0.0651	0.103	0.0504	60
Juan Ramirez	Hispanic	0.815	0.754	1.723	1.554	0.0538	0.0744	0.0878	0.0485	65
Javier Rodriguez	Hispanic	0.815	0.759	1.667	1.667	0.0587	0.0699	0.0989	0.0534	54

Table S1: Average Characteristics for Individual Names, Sorted by Identity and Probability of a Correct Response

Name	Identity	Pr. Correct	Pr. Citizen	Income (1-3)	Education (1-4)	Citizen SE	Income SE	Education SE	Correct SE	N
Miguel Ramirez	Hispanic	0.812	0.750	1.583	1.646	0.0632	0.0885	0.113	0.0569	48
Alejandro Rodriguez	Hispanic	0.810	0.724	1.862	1.862	0.0592	0.0902	0.122	0.0519	58
Javier Hernandez	Hispanic	0.807	0.719	1.895	1.719	0.0600	0.0818	0.114	0.0527	57
Jorge Lopez	Hispanic	0.803	0.833	1.727	1.877	0.0462	0.0823	0.113	0.0493	66
Salvador Torres	Hispanic	0.803	0.704	1.746	1.775	0.0545	0.0687	0.0902	0.0476	71
Alejandro Torres	Hispanic	0.803	0.724	1.693	1.720	0.0516	0.0629	0.0941	0.0460	76
Alejandro Sanchez	Hispanic	0.800	0.727	1.745	1.685	0.0606	0.0943	0.0983	0.0544	55
Hector Sanchez	Hispanic	0.797	0.814	1.847	1.695	0.0511	0.0718	0.0913	0.0529	59
Juan Martinez	Hispanic	0.796	0.741	1.519	1.722	0.0602	0.0782	0.104	0.0553	54
Miguel Hernandez	Hispanic	0.795	0.685	1.712	1.767	0.0547	0.0742	0.104	0.0476	73
Julio Torres	Hispanic	0.794	0.765	1.761	1.838	0.0518	0.0739	0.104	0.0494	68
Salvador Garcia	Hispanic	0.794	0.667	1.540	1.587	0.0599	0.0709	0.0977	0.0514	63
Jorge Gonzalez	Hispanic	0.793	0.707	1.552	1.621	0.0603	0.0822	0.101	0.0537	58
Alejandro Hernandez	Hispanic	0.792	0.679	1.868	1.887	0.0647	0.0810	0.113	0.0562	53
Juan Lopez	Hispanic	0.792	0.811	1.925	1.925	0.0543	0.0927	0.126	0.0562	53
Juan Perez	Hispanic	0.792	0.667	1.771	1.875	0.0688	0.0855	0.114	0.0592	48
Jorge Perez	Hispanic	0.788	0.673	1.769	1.750	0.0657	0.0806	0.109	0.0572	52
Guadalupe Hernandez	Hispanic	0.788	0.727	1.652	1.742	0.0552	0.0732	0.109	0.0507	66
Jorge Hernandez	Hispanic	0.787	0.656	1.705	1.852	0.0613	0.0821	0.109	0.0529	61
Alejandro Garcia	Hispanic	0.787	0.770	1.852	1.951	0.0543	0.0900	0.116	0.0529	61
Guadalupe Ramirez	Hispanic	0.787	0.600	1.600	1.680	0.0569	0.0684	0.0933	0.0476	75
Julio Rodriguez	Hispanic	0.786	0.679	1.707	1.750	0.0513	0.0637	0.0926	0.0450	84
Jesus Sanchez	Hispanic	0.785	0.769	1.708	1.766	0.0527	0.0812	0.109	0.0514	65
Miguel Martinez	Hispanic	0.783	0.783	1.717	1.695	0.0536	0.0827	0.106	0.0536	60
Miguel Garcia	Hispanic	0.783	0.807	1.831	1.904	0.0436	0.0637	0.0884	0.0455	83
Salvador Martinez	Hispanic	0.780	0.610	1.729	1.797	0.0640	0.0758	0.113	0.0544	59
Alejandro Martinez	Hispanic	0.778	0.746	1.794	1.921	0.0553	0.0789	0.109	0.0528	63
Salvador Hernandez	Hispanic	0.775	0.676	1.662	1.662	0.0559	0.0775	0.106	0.0499	71
Guadalupe Lopez	Hispanic	0.771	0.646	1.646	1.708	0.0698	0.101	0.130	0.0613	48
Javier Ramirez	Hispanic	0.771	0.812	1.708	1.792	0.0569	0.0891	0.123	0.0613	48
Javier Lopez	Hispanic	0.770	0.721	1.738	1.738	0.0579	0.0806	0.0932	0.0543	61
Guadalupe Gonzalez	Hispanic	0.767	0.548	1.603	1.575	0.0587	0.0668	0.0913	0.0498	73
Javier Garcia	Hispanic	0.767	0.717	1.867	2.033	0.0587	0.0839	0.121	0.0551	60
Jesus Hernandez	Hispanic	0.767	0.667	1.717	1.800	0.0614	0.0755	0.106	0.0551	60
Juan Hernandez	Hispanic	0.762	0.730	1.629	1.714	0.0564	0.0699	0.105	0.0541	63
Hector Lopez	Hispanic	0.760	0.707	1.773	1.703	0.0529	0.0796	0.101	0.0496	75
Miguel Gonzalez	Hispanic	0.754	0.789	1.596	1.719	0.0545	0.0786	0.111	0.0575	57
Jesus Rodriguez	Hispanic	0.754	0.656	1.590	1.574	0.0613	0.0753	0.0858	0.0556	61
Hector Hernandez	Hispanic	0.754	0.738	1.672	1.770	0.0568	0.0729	0.106	0.0556	61

Table S1: Average Characteristics for Individual Names, Sorted by Identity and Probability of a Correct Response

Name	Identity	Pr. Correct	Pr. Citizen	Income (1-3)	Education (1-4)	Citizen SE	Income SE	Education SE	Correct SE	N
Juan Garcia	Hispanic	0.754	0.826	1.623	1.696	0.0460	0.0718	0.0975	0.0523	69
Alejandro Gonzalez	Hispanic	0.754	0.812	1.725	1.797	0.0474	0.0769	0.102	0.0523	69
Juan Rodriguez	Hispanic	0.753	0.792	1.753	1.805	0.0465	0.0671	0.0961	0.0495	77
Jesus Gonzalez	Hispanic	0.750	0.750	1.662	1.779	0.0529	0.0682	0.100	0.0529	68
Jorge Sanchez	Hispanic	0.750	0.734	1.625	1.794	0.0556	0.0686	0.107	0.0546	64
Miguel Perez	Hispanic	0.746	0.672	1.761	1.591	0.0578	0.0769	0.0890	0.0536	67
Guadalupe Sanchez	Hispanic	0.746	0.571	1.556	1.619	0.0628	0.0777	0.112	0.0553	63
Salvador Rodriguez	Hispanic	0.742	0.773	1.788	1.894	0.0520	0.0733	0.117	0.0542	66
Jorge Garcia	Hispanic	0.741	0.741	1.741	1.862	0.0580	0.0836	0.129	0.0580	58
Guadalupe Garcia	Hispanic	0.738	0.677	1.554	1.723	0.0585	0.0761	0.104	0.0549	65
Julio Martinez	Hispanic	0.738	0.723	1.662	1.769	0.0559	0.0736	0.102	0.0549	65
Miguel Rodriguez	Hispanic	0.737	0.754	1.789	1.947	0.0575	0.0740	0.131	0.0588	57
Jesus Garcia	Hispanic	0.736	0.660	1.736	1.792	0.0657	0.0858	0.119	0.0611	53
Julio Lopez	Hispanic	0.736	0.698	1.774	1.717	0.0637	0.0919	0.0985	0.0611	53
Juan Torres	Hispanic	0.735	0.779	1.779	1.627	0.0507	0.0657	0.0762	0.0539	68
Guadalupe Perez	Hispanic	0.735	0.603	1.603	1.761	0.0598	0.0699	0.111	0.0539	68
Miguel Torres	Hispanic	0.732	0.714	1.750	1.782	0.0609	0.0775	0.112	0.0597	56
Salvador Sanchez	Hispanic	0.732	0.714	1.786	1.778	0.0609	0.0834	0.111	0.0597	56
Guadalupe Torres	Hispanic	0.731	0.673	1.673	1.882	0.0657	0.0811	0.118	0.0621	52
Hector Martinez	Hispanic	0.725	0.745	1.627	1.735	0.0616	0.0884	0.116	0.0631	51
Juan Sanchez	Hispanic	0.719	0.641	1.734	1.938	0.0605	0.0747	0.107	0.0566	64
Javier Torres	Hispanic	0.714	0.743	1.681	1.729	0.0526	0.0636	0.0908	0.0544	70
Jorge Martinez	Hispanic	0.710	0.841	1.710	1.824	0.0444	0.0688	0.0912	0.0550	69
Julio Hernandez	Hispanic	0.710	0.758	1.689	1.726	0.0548	0.0794	0.101	0.0581	62
Julio Sanchez	Hispanic	0.696	0.786	1.696	1.732	0.0553	0.0803	0.113	0.0620	56
Javier Perez	Hispanic	0.683	0.698	1.619	1.635	0.0583	0.0731	0.109	0.0591	63
Hector Perez	Hispanic	0.676	0.746	1.732	2.029	0.0520	0.0850	0.120	0.0559	71
Jorge Torres	Hispanic	0.672	0.781	1.766	1.828	0.0521	0.0695	0.103	0.0592	64
Salvador Perez	Hispanic	0.672	0.688	1.734	1.688	0.0584	0.0747	0.0887	0.0592	64
Jorge Ramirez	Hispanic	0.657	0.800	1.729	1.800	0.0482	0.0759	0.0901	0.0571	70
Jesus Torres	Hispanic	0.574	0.721	1.852	1.902	0.0579	0.0804	0.119	0.0638	61
Hector Torres	Hispanic	0.548	0.839	1.839	1.935	0.0471	0.0803	0.108	0.0637	62
Thomas Wagner	White	0.931	0.911	2.030	2.110	0.0285	0.0516	0.0777	0.0254	101
Richard Hoffman	White	0.925	0.946	2.312	2.419	0.0235	0.0611	0.0896	0.0275	93
Mary Meyer	White	0.911	0.978	1.822	1.911	0.0156	0.0624	0.0784	0.0302	90
Richard Schmidt	White	0.905	0.947	2.160	2.316	0.0230	0.0509	0.0876	0.0302	95
Thomas Snyder	White	0.905	0.976	1.940	2.060	0.0167	0.0685	0.0836	0.0322	84
Michael Wagner	White	0.900	0.910	1.970	2.180	0.0288	0.0559	0.0757	0.0302	100
Robert Snyder	White	0.890	0.954	2.073	2.130	0.0201	0.0593	0.0768	0.0301	109

Table S1: Average Characteristics for Individual Names, Sorted by Identity and Probability of a Correct Response

Name	Identity	Pr. Correct	Pr. Citizen	Income (1-3)	Education (1-4)	Citizen SE	Income SE	Education SE	Correct SE	N
Mark Meyer	White	0.886	0.990	2.077	2.086	0.00952	0.0575	0.0743	0.0312	105
David Ryan	White	0.886	0.971	1.943	2	0.0163	0.0503	0.0754	0.0312	105
Mark Hoffman	White	0.886	0.971	2.152	2.192	0.0163	0.0538	0.0724	0.0312	105
Mary Ryan	White	0.884	0.979	1.904	1.926	0.0148	0.0547	0.0737	0.0330	95
John Schmidt	White	0.882	0.925	2.054	2.183	0.0275	0.0538	0.0716	0.0337	93
William Schmidt	White	0.874	0.910	2.081	2.182	0.0273	0.0588	0.0788	0.0317	111
Robert Hoffman	White	0.873	0.961	2.049	2.216	0.0193	0.0613	0.0888	0.0332	102
James Hoffman	White	0.873	0.961	2.088	2.265	0.0193	0.0625	0.0800	0.0332	102
Mark Hansen	White	0.872	0.968	2.021	2.128	0.0182	0.0566	0.0760	0.0346	94
John Ryan	White	0.870	0.963	1.944	2.019	0.0183	0.0540	0.0818	0.0325	108
William Snyder	White	0.870	0.957	2.054	2.198	0.0214	0.0644	0.0769	0.0353	92
Richard Meyer	White	0.865	0.958	2.083	2.116	0.0205	0.0547	0.0845	0.0351	96
Thomas Meyer	White	0.864	0.938	1.988	2.235	0.0269	0.0669	0.0982	0.0383	81
Thomas Schmidt	White	0.864	0.913	2.029	2.146	0.0280	0.0576	0.0821	0.0339	103
John Snyder	White	0.860	0.974	1.921	2.009	0.0151	0.0516	0.0770	0.0327	114
David Snyder	White	0.859	0.939	1.960	2.202	0.0241	0.0608	0.0812	0.0352	99
David Wagner	White	0.857	0.933	1.990	2.190	0.0245	0.0555	0.0767	0.0343	105
David Hoffman	White	0.854	0.948	2.125	2.312	0.0228	0.0539	0.0867	0.0362	96
John Meyer	White	0.852	0.969	2.142	2.157	0.0154	0.0509	0.0606	0.0315	128
Thomas Olson	White	0.850	0.975	2.075	2.101	0.0176	0.0636	0.0756	0.0402	80
Mark Wagner	White	0.849	0.935	2.033	2.258	0.0256	0.0523	0.0836	0.0373	93
James Ryan	White	0.849	0.953	1.930	2.023	0.0228	0.0614	0.0827	0.0389	86
Mary Snyder	White	0.848	0.946	1.884	1.964	0.0214	0.0549	0.0760	0.0341	112
William Wagner	White	0.848	0.978	2.022	2.143	0.0153	0.0578	0.0835	0.0377	92
Michael Hoffman	White	0.848	0.957	2.087	2.228	0.0214	0.0631	0.0916	0.0377	92
William Hoffman	White	0.848	0.952	2.133	2.248	0.0209	0.0591	0.0810	0.0352	105
David Schmidt	White	0.846	0.901	2.055	2.253	0.0315	0.0632	0.0850	0.0380	91
David Meyer	White	0.846	0.942	2.048	2.135	0.0230	0.0500	0.0749	0.0356	104
James Snyder	White	0.845	0.964	1.964	2.202	0.0204	0.0547	0.0843	0.0397	84
James Schmidt	White	0.845	0.922	2.010	2.175	0.0265	0.0543	0.0798	0.0359	103
Mary Hoffman	White	0.844	0.938	2.042	2.135	0.0248	0.0627	0.0792	0.0373	96
Thomas Larson	White	0.840	0.953	1.962	2.114	0.0207	0.0500	0.0733	0.0358	106
Mark Schmidt	White	0.839	1	2.032	2.087	0	0.0561	0.0736	0.0383	93
Thomas Hansen	White	0.838	0.905	1.962	2.115	0.0288	0.0523	0.0687	0.0361	105
John Hoffman	White	0.838	0.937	2.117	2.198	0.0232	0.0491	0.0711	0.0351	111
David Olson	White	0.837	0.904	1.951	2.067	0.0290	0.0607	0.0713	0.0364	104
William Larson	White	0.835	0.953	2.129	2.153	0.0231	0.0599	0.0909	0.0405	85
Richard Olson	White	0.833	0.933	2.111	2.178	0.0264	0.0621	0.0815	0.0395	90
John Olson	White	0.828	0.939	1.949	2.040	0.0241	0.0505	0.0657	0.0381	99

Table S1: Average Characteristics for Individual Names, Sorted by Identity and Probability of a Correct Response

Name	Identity	Pr. Correct	Pr. Citizen	Income (1-3)	Education (1-4)	Citizen SE	Income SE	Education SE	Correct SE	N
Richard Ryan	White	0.828	0.935	2.032	2.258	0.0256	0.0561	0.0793	0.0393	93
Robert Wagner	White	0.826	0.945	2.101	2.284	0.0219	0.0611	0.0794	0.0365	109
Mary Schmidt	White	0.824	0.956	1.879	2.022	0.0216	0.0560	0.0782	0.0401	91
Thomas Hoffman	White	0.823	0.938	2.018	2.241	0.0228	0.0470	0.0637	0.0361	113
Thomas Ryan	White	0.820	0.934	1.869	1.943	0.0225	0.0533	0.0619	0.0350	122
Mark Ryan	White	0.819	0.936	1.947	2	0.0253	0.0573	0.0794	0.0399	94
John Larson	White	0.819	0.943	2.038	2.257	0.0228	0.0505	0.0766	0.0378	105
Mark Snyder	White	0.819	0.933	2.048	2.152	0.0245	0.0586	0.0821	0.0378	105
Robert Ryan	White	0.818	0.936	1.936	2.083	0.0234	0.0521	0.0750	0.0369	110
Robert Meyer	White	0.817	0.942	2	2.231	0.0230	0.0511	0.0776	0.0381	104
Michael Meyer	White	0.816	0.961	1.913	1.951	0.0191	0.0500	0.0644	0.0384	103
Michael Snyder	White	0.815	0.946	1.989	2.109	0.0238	0.0546	0.0699	0.0407	92
Mary Olson	White	0.815	0.957	1.924	2.054	0.0214	0.0562	0.0714	0.0407	92
Richard Wagner	White	0.814	0.866	2.021	2.165	0.0348	0.0586	0.0812	0.0397	97
Mary Wagner	White	0.814	0.959	1.907	1.979	0.0203	0.0623	0.0854	0.0397	97
Robert Larson	White	0.814	0.920	2.018	2.144	0.0256	0.0576	0.0838	0.0368	113
David Larson	White	0.812	0.961	2	2.094	0.0172	0.0444	0.0632	0.0346	128
John Hansen	White	0.804	0.957	1.978	2.066	0.0214	0.0618	0.0809	0.0416	92
Mark Larson	White	0.804	0.971	1.971	2.078	0.0168	0.0530	0.0706	0.0395	102
Robert Olson	White	0.802	0.950	2.089	2.297	0.0217	0.0547	0.0839	0.0399	101
Mary Hansen	White	0.802	0.953	1.887	2.142	0.0207	0.0525	0.0798	0.0389	106
William Meyer	White	0.800	0.945	2.027	2.173	0.0218	0.0508	0.0706	0.0383	110
Richard Larson	White	0.798	0.945	2.028	2.110	0.0219	0.0560	0.0838	0.0386	109
Robert Hansen	White	0.798	0.933	2.078	2.272	0.0247	0.0580	0.0745	0.0396	104
Robert Schmidt	White	0.796	0.922	2	2.243	0.0265	0.0557	0.0822	0.0399	103
Mark Olson	White	0.789	0.881	2.009	2.018	0.0312	0.0561	0.0749	0.0393	109
David Hansen	White	0.788	0.942	2.010	2.087	0.0230	0.0555	0.0810	0.0402	104
Michael Schmidt	White	0.788	0.899	2.030	2.283	0.0304	0.0527	0.0704	0.0413	99
Michael Hansen	White	0.787	0.957	2	2.106	0.0209	0.0524	0.0748	0.0424	94
James Meyer	White	0.786	0.981	2.010	2.165	0.0137	0.0488	0.0619	0.0406	103
William Ryan	White	0.786	0.939	2.020	2.173	0.0243	0.0598	0.0821	0.0417	98
James Olson	White	0.781	0.958	1.979	1.958	0.0205	0.0491	0.0644	0.0424	96
William Hansen	White	0.774	0.946	2.086	2.312	0.0235	0.0624	0.0891	0.0436	93
Michael Ryan	White	0.764	0.966	1.933	2.022	0.0192	0.0635	0.0766	0.0453	89
Richard Hansen	White	0.763	0.959	2.134	2.247	0.0203	0.0500	0.0803	0.0434	97
Richard Johnston	White	0.761	0.978	2	2.133	0.0153	0.0656	0.0839	0.0447	92
Mary Johnston	White	0.758	0.968	1.905	2.021	0.0180	0.0520	0.0748	0.0442	95
John Wagner	White	0.757	0.907	2.009	2.093	0.0283	0.0488	0.0697	0.0417	107
Richard Snyder	White	0.756	0.949	2.026	2.141	0.0251	0.0631	0.0889	0.0489	78

Table S1: Average Characteristics for Individual Names, Sorted by Identity and Probability of a Correct Response

Name	Identity	Pr. Correct	Pr. Citizen	Income (1-3)	Education (1-4)	Citizen SE	Income SE	Education SE	Correct SE	N
Mark Johnston	White	0.755	0.968	1.851	1.926	0.0182	0.0544	0.0729	0.0446	94
David Johnston	White	0.755	0.989	2.011	2.191	0.0106	0.0576	0.0818	0.0446	94
James Larson	White	0.753	0.903	2.011	2.055	0.0308	0.0654	0.0832	0.0450	93
John Johnston	White	0.748	0.942	1.853	1.922	0.0232	0.0564	0.0801	0.0430	103
Mary Larson	White	0.747	0.977	1.977	2.116	0.0162	0.0589	0.0882	0.0469	87
James Wagner	White	0.742	0.978	1.944	2.135	0.0158	0.0626	0.0892	0.0467	89
Michael Larson	White	0.738	0.938	1.913	2.087	0.0272	0.0539	0.0716	0.0495	80
Michael Olson	White	0.730	0.926	2.041	2.139	0.0238	0.0442	0.0672	0.0404	122
James Hansen	White	0.724	0.966	1.884	1.977	0.0197	0.0559	0.0766	0.0482	87
William Olson	White	0.717	0.935	1.935	2.043	0.0259	0.0553	0.0740	0.0472	92
Thomas Johnston	White	0.698	0.938	1.905	1.844	0.0248	0.0636	0.0774	0.0471	96
William Johnston	White	0.690	0.966	2	2.155	0.0170	0.0519	0.0780	0.0431	116
Robert Johnston	White	0.680	0.940	2	2.090	0.0239	0.0532	0.0767	0.0469	100
Michael Johnston	White	0.624	0.976	1.894	2.035	0.0165	0.0580	0.0953	0.0529	85
James Johnston	White	0.613	0.950	1.875	2.150	0.0245	0.0626	0.0943	0.0548	80
